# Activation of the periaqueductal gray controls respiratory output through a distributed brain network

**DOI:** 10.3389/fphys.2025.1516771

**Published:** 2025-01-22

**Authors:** Mitchell Prostebby, Jashan Saini, Vivian Biancardi, Clayton T. Dickson, Silvia Pagliardini

**Affiliations:** ^1^ Neuroscience and Mental Health Institute, University of Alberta, Edmonton, AB, Canada; ^2^ Department of Physiology, University of Alberta, Edmonton, AB, Canada; ^3^ Women and Children’s Health Research Institute, University of Alberta, Edmonton, AB, Canada; ^4^ Department of Psychology, University of Alberta, Edmonton, AB, Canada

**Keywords:** periaqueductal gray, optogenetics, respiratory control, respiratory reset, PreBotzinger complex

## Abstract

**Introduction:**

The periaqueductal gray (PAG) has been previously established to play a key role in producing the vital changes in respiration occurring in response to threat. However, it is not fully understood how PAG activation alters the ongoing respiratory output, nor it is understood which pathways mediate these effects, as several regions have been previously identified to influence respiratory activity.

**Methods:**

We used optogenetic tools in conjunction with EMG recordings of inspiratory and expiratory musculature to determine how PAG activation on short (250 ms) and longer (10–15 s) timescales alters respiratory muscle activity. Through cFOS mapping, we also identified key downstream brain regions which were likely modulated by PAG activation including the preBötzinger Complex (preBötC) and the lateral parafacial area (pFL). We then stimulated PAG terminals in those regions to determine whether their activity can account for the observed effects of PAG stimulation.

**Results:**

Directly stimulating the PAG resulted in prominent changes to all recorded muscle activities and reset the breathing rhythm in either a phase-independent or phase-dependent manner. In contrast, stimulating PAG terminals in either preBötC or pFL with long or shorter timescale stimuli could not completely replicate the effects of direct PAG stimulation and also did not produce any respiratory reset.

**Conclusions:**

Our results show that the effects of PAG activity on respiration are not mediated solely by PAG inputs to either the preBötC or pFL and more likely involve integration across a larger network of brainstem areas.

## Introduction

Respiration is a vital function which is mediated by a complex set of neural interactions that maintain the breathing cycle throughout the stresses we encounter in our daily lives. This includes modulating inspiratory and expiratory musculature in response to a perceived internal or external stressor. The periaqueductal gray (PAG) has long been implicated as a midbrain area critically important in integrating and promoting defensive behaviors, vocalization, reward seeking, fear learning as well as cardiovascular and respiratory changes underlying these responses (Benarroch, 2012; Bandler and Shipley, 1994; Fanselow et al., 1991; Jürgens, 1998; Jürgens, 2002; Dampney et al., 2013; Motta et al., 2017; Zhang et al., 2024; Carrive, 1993). Specifically, the columns of the PAG are known to receive inputs from the forebrain (medial prefrontal cortex, hypothalamus, amygdala, insular cortex), nociceptive pathways in the dorsal spinal cord and the spinal trigeminal nucleus, as well as vagal inputs through the nucleus of the solitary tract, and a dense catecholaminergic innervation from various structures in the brainstem (Benarroch, 2012; Dampney et al., 2013; Zhang et al., 2024; Trevizan-Baú et al., 2021a; Trevizan-Baú et al., 2021b; Ma et al., 2023). In return, the PAG projects to thalamus and hypothalamus as well as pontine and brainstem structures involved in motor and autonomic control and nociception (Benarroch, 2012; Dampney et al., 2013; Bandler et al., 1991).

Stimulation of the PAG via chemical injection or direct electrical stimulation induces changes in arterial blood pressure, heart rate, respiration rate, and respiratory muscle activity that are column specific. In particular, stimulation of the dorsolateral and lateral columns is associated with sympathoexcitation and increase in respiratory rate and vocalization, while ventrolateral activation is responsible for sympathoinhibitory responses and respiratory depression (Huang et al., 2000; Zhang et al., 2007; Zhang et al., 2005; Zhang et al., 2009; Subramanian et al., 2008; Iigaya et al., 2010; Bandler and Carrive, 1988).

The pathway which links the respiratory effects with PAG output, however, is complex and not fully understood. Tracing studies suggest the existence of both uni- and bi-directional projections with the preBötzinger Complex (preBötC, the inspiratory rhythm oscillator located in the medulla), in addition to other brainstem respiratory-related structures, such as the lateral parafacial (pFL, responsible for the generation of active expiration (Pagliardini et al., 2011; Del Negro et al., 2018), the Kölliker-Fuse (KF), the parabrachial nucleus (PB), the nucleus of the solitary tract (NTS), the nucleus retroambiguus (NRA), the locus coeruleus (LC), the post-inspiratory complex (Pico) and the retrotrapezoid nucleus (RTN) (Zhang et al., 2024; Trevizan-Baú et al., 2021b; Tan et al., 2010; Biancardi et al., 2021; Ennis et al., 1997; van Bockstaele et al., 1991; Biancardi et al., 2023; Oliveira et al., 2021). These connections are presumed to be responsible for the PAG effects on respiration that are linked to emotional or painful responses, although indirect projections through the hypothalamus have also been suggested (Iigaya et al., 2010). We aimed to investigate whether the noted effects of the PAG stimulation could be primarily attributed to its direct connections with the respiratory rhythmogenic areas (preBötC and pFL). To do so, we stimulated the PAG and its projections directly into preBötC and pFL using optogenetics. By leveraging the temporal specificity of these tools we aimed to determine whether stimulation during specific phases of the respiratory rhythm could interfere with ongoing oscillatory activity. We hypothesized that PAG activation on a broad timescale would potentiate both inspiratory and expiratory muscle contractions and increase the rate of breathing via the projections to the major inspiratory and expiratory oscillators in the preBӧtC and the pFL. Moreover, short timescale stimulation of PAG projections to the preBӧtC was hypothesized to reset the respiratory rhythm in a phase-independent manner as demonstrated by previous investigations that focused on direct stimulation of preBötC neurons (Alsahafi et al., 2015; Vann et al., 2018). In order to identify brain regions activated by the PAG stimulation we also assessed expression of cFOS in various structures involved in respiratory control that have been proposed to receive inputs from the PAG.

Our results showed that long duration lateral/ventrolateral PAG photostimulation profoundly increased respiratory frequency, while potentiating respiratory activity. While brief pulses of stimulation in PAG induced a consistent phase advance of the subsequent respiratory cycle, the stimulation of the presynaptic terminals in preBӧtC or pFL could not fully mimic the effects obtained with PAG stimulation, although increased cFOS expression was observed in these areas involved in respiratory control. Photostimulation delivered at the level of the preBötC increased inspiratory muscle activity and respiratory rate with no respiratory reset while photostimulation in the pFL did not induce reset or active expiration, although some potentiation of genioglossus muscle activity was observed. These results suggest that perturbation of ongoing respiratory rhythm requires an extended and distributed network of connections from the PAG, likely to a variety of respiratory centers.

## Methods

### Ethics approval

All experimental protocols were approved by the University of Alberta Animal Policy and Welfare Committee (animal use protocol # 461) according to the research ethics standards set by the Canadian Council on Animal Care.

### Viral injection into the periaqueductal gray

Optogenetic control was established via viral infection of the PAG using adeno-associated virus (AAV, serotype 2/5) expressing channelrhodopsin-2 (ChR2) and enhanced yellow fluorescent protein (eYFP), driven by human synapsin promoter (hSYN) (hSYN-ChR2-eYFP; 4 × 10^12^ molecules/mL; University of North Carolina Virus Vector Core, Chapel Hill, NC, United States). Control experiments were run using naïve rats. Injections of AAV serotype 9 expressing identical constructs were also performed to validate our results with viruses showing preferential axonal expression of opsins (Jackman et al., 2014) (1 × 10^13^ molecules/mL; Addgene, Watertown, MA, United States). Unilateral viral injections were made into the lateral subdivision of the periaqueductal gray (L PAG) in adult, male Sprague Dawley rats (250–300 g) (n = 32, see Exclusion Criteria). Anaesthesia was induced via i. p injections of ketamine-xylazine (90–10 mg/kg) before rats were positioned prone on a stereotaxic apparatus. A midline incision was made to expose the skull and stereotaxic coordinates were assessed relative to bregma to target the lateral PAG (−7.8 mm RC, +2.73 mm ML). A small hole was made on the skull above the target before the micropipette was slowly advanced 4.6 mm from the surface of the brain at an angle of 22°. Once the target was reached, the viral solution (∼300 nL) was delivered via back-pressure injection (20 p. s.i., 25–50 ms pulses; PicoSpritzer III, Parker, ON, Canada). Micropipette retraction was delayed for 5 min to ensure prevention of solution backflow and skin and muscles were sutured back. Body temperature was maintained at 37°C throughout the surgical procedure using a servo-controlled heating pad (Harvard Apparatus, Holliston, MA, United States). Metacam analgesic was administered 1 h prior to surgery (2 mg/kg). Following surgery, rats received local anaesthetic bupivacaine (0.2 mL, s. c.) and were treated with metacam analgesic for 3 days. Food and water were delivered *ad libitum* throughout the 3-week recovery period in accordance with our animal use protocol.

### Optogenetic stimulation protocol

Three weeks after viral injection, rats were prepared for surgery and optogenetic stimulation. Anaesthesia was initially induced using 2% isoflurane while the femoral vein was cannulated, after which urethane (1.5–1.7 g/kg body weight) was delivered intravenously to maintain a surgical plane of anaesthesia. Coupled EMG electrodes (CoonerWire, Chatsworth, CA, United States) were then implanted into the diaphragm (DIA), genioglossus (GG), and oblique abdominal (ABD) muscles and a bilateral vagotomy was performed. EMG signals were amplified at 1,000x (AM Systems, Sequim, WA, United States), digitized by a Powerlab 16/35 acquisition system (ADInstruments, Colorado Springs, CO, United States) and sampled at 1 kHz.

Rats were positioned on a stereotaxic frame with bregma and lambda flat. After a small hole was drilled to access the brain, an optic fibre was carefully advanced into the PAG above the viral injection (−7.8 mm RC, +2.73 mm ML, −4.2 to −4.6 mm DV). The stimulation protocol consisted of laser pulses at 473 nm (IkeCool, Anaheim, CA, United States and Shanghai DreamLaser Technology, Shanghai, China) set to 3 different modes: 10 s pulses, high frequency train (20 ms pulses at 20Hz, 200–300 repeats), and brief pulses of 250 ms (0.1Hz, 150 repeats) delivered with a Ø200 µm Core optic fiber (0.22 NA; Thorlabs Inc., Newton, NJ, United States). Each stimulus was delivered via LabChart8 software (ADInstruments, Colorado Springs, CO, United States) and all laser sources were calibrated to emit light at 10–12 mW.

We also performed photostimulation of PAG terminals in the preBötC (N = 20, see Exclusion Criteria) and pFL (n = 7, see Exclusion Criteria), either prior to or following PAG stimulation. Rats were positioned on the stereotaxic apparatus such that bregma was 5 mm below lambda. These sites were accessed from the dorsal aspect of the brainstem by displacing the neck muscles and the occipital bone. The optic fibre was advanced to the top of preBötC using the following coordinates: +0.9 mm RC, +2.0 mm ML, and −2.6 mm DV relative to the obex. In case of bilateral stimulation of the preBötC (N = 9), an additional optic fibre of the same caliber was positioned −2.0 mm ML contralaterally to the obex at the same RC and DV coordinates as in unilateral stimulation. Bilateral stimulation of the pFL (n = 7) was performed either before or after stimulating the preBötC at coordinates +2.0 mm RC, ±2.2 mm ML, and −3.4DV relative to obex. At the end of the experiment, rats were transcardially perfused with phosphate buffered, PB, followed by 4% paraformaldehyde in PB, and their brains were processed according to the histological preparations described below.

### Expression of cFos in the respiratory network following PAG stimulation

In 7 rats, we restricted our experimental protocol to stimulating only the PAG in order to compare the expression of cFos, a marker of elevated neuronal activity in the respiratory network of both hSYN-ChR2-eYFP and naïve rats. In this set of experiments, the laser stimulus consisted of high frequency trains (25 ms pulses, 20 Hz, 300 repeats) interleaved with 20 s of delay, repeated for a total duration of 15 min (Figure 8A). Rats were then let to rest for at least 30–45 min and then transcardially perfused with phosphate buffer followed by 4% paraformaldehyde in phosphate buffer, and their brains collected for histological processing as described below.

### Histology

**FIGURE 1:**
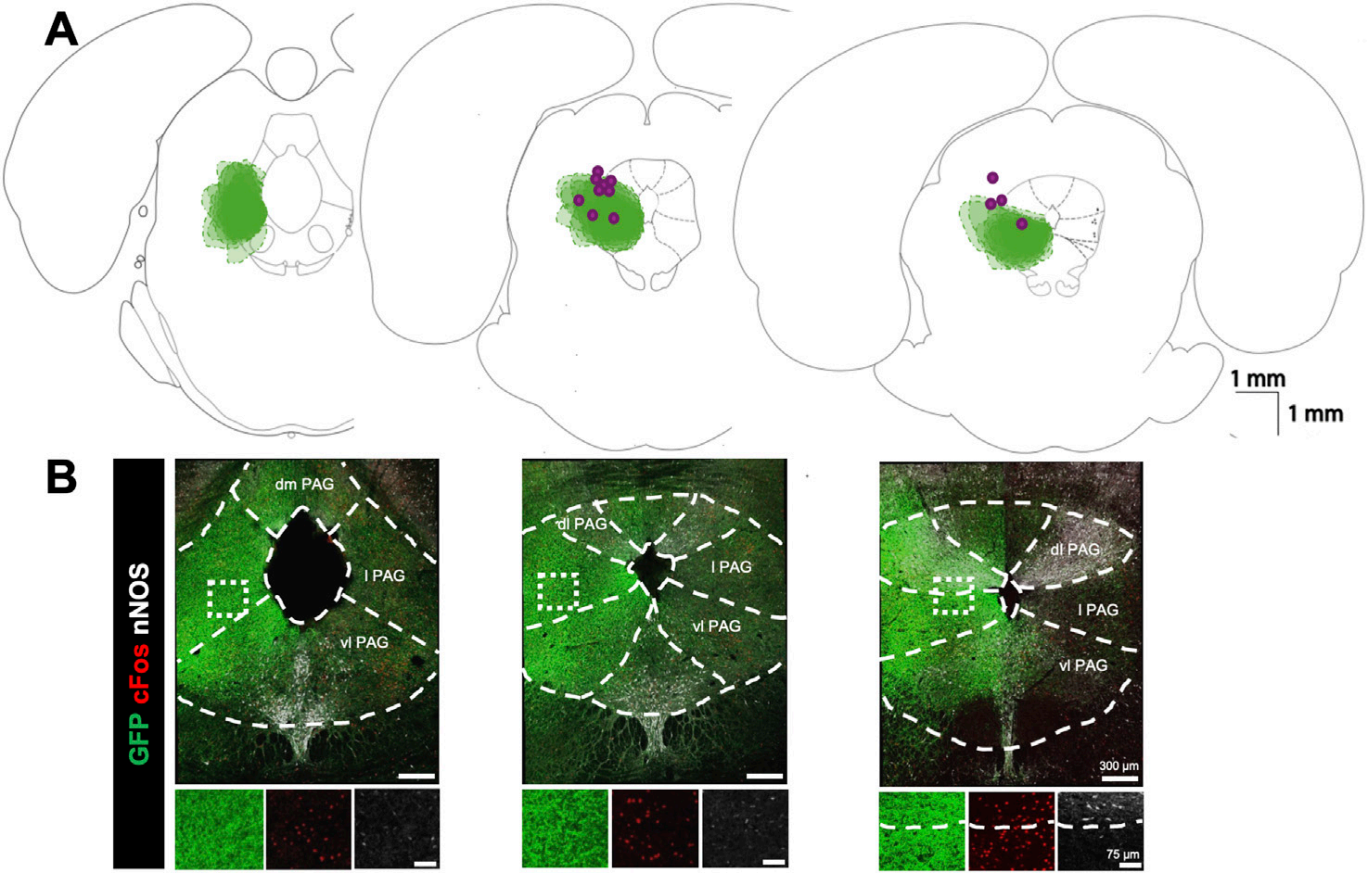
Viral expression profile for optogenetic stimulation of the lateral PAG: **(A)** Expression map for optogenetic virus and optic fibre locations. Green clouds indicate the extent of GFP expression in three coronal sections arranged from caudal (left) to rostral (right) along the core of the PAG. Clouds from each experiment are overlaid (n = 13). Purple dots indicate optic fibre tip locations. **(B)** Examples of immunofluorescence staining in the corresponding sections above in A for GFP (green), cFOS (red), and nNOS (white). Dashed lines demarcate the boundaries for PAG subdivisions based on nNOS fluorescence and histological landmarks (dm, dorsomedial; dl, dorsolateral; l lateral; vl, ventrolateral). Insets below show high power magnification of immunostained tissue in separate channels. Right panel highlights boundaries between lateral and dorsolateral PAG based on nNOS staining.

The perfused brains were cryoprotected in 30% sucrose solution, frozen and sectioned at 50 μm (Leica CM1950 Cryostat) for free-floating immunohistochemistry. Brain slices in the midbrain and brainstem were selected 300 μm apart and washed with phosphate buffer saline (PBS) before incubating for 60 min with 0.3% Triton X-100 and 10% normal donkey serum (NDS) in PBS to ensure optimal antibody penetration and reduce non-specific staining. Sections of the PAG were incubated overnight (12–16 h) at room temperature with primary antibody solutions containing chicken polyclonal IgY antibody anti-GFP (1:500; Aves Labs Cat# GFP-1010, RRID: AB_2307313), and mouse anti-neuronal Nitric Oxide Synthase (nNOS, 1:500; Santa Cruz Biotechnology Cat# sc-5302, RRID: AB_626757). Primary antibody solutions for immunodetection of markers of neuronal activity included chicken anti-GFP (1:500), mouse anti-Neuronal Nuclei (NeuN, 1:500; Millipore Sigma Cat#MAB377, RRID: AB_2298772), and rabbit anti-c-FOS (1:300; Millipore Sigma Cat#PC05-100UG, RRID: AB_565444). All primary antibody solutions were diluted with 1% NDS and 0.3% Triton X-100. Sections were washed with PBS the following day before a 2 h incubation with appropriate secondary antibodies conjugated with fluorescent probes (Cy2-conjugated donkey anti-chicken IgY (1:200; Cat# 703–225–155, RRID:AB_2340370); Cy5-conjugated donkey anti-mouse IgG (1:200; Cat# 715–175–150, RRID:AB_2340819); AF350-conjugated donkey anti-rabbit (1:150; Cat# A10039, RRID:AB_2534015) diluted in PBS and 1%NDS. A final PBS wash was performed before mounting slides and coverslipping them with Fluorsave solution (Millipore, Billerica, MA, United States). Slides were imaged using a EVOS FL Epifluorescent microscope (Life Technologies/ThermoFisher Scientific, Waltham, MA, United States) under 4x and 10x magnification to assess extension of viral expression and counting of cFos + cells in the subdivision of PAG and in selected structures of the respiratory network (parabrachial nuclei, PBN; locus coeruleus, LC; nucleus of the solitary tract, NTS, ventral respiratory column, VRC) as described in (Biancardi et al., 2021). For illustration purposes (Figures 1, 5), images of brainstem areas infected by the virus were taken using a Leica TCS SP8 Laser Scanning Confocal Microscope in the Cell Imaging Core facility at the University of Alberta.

### cFos expression analysis

To illustrate the status of activation within the different PAG subregions, we analyzed and counted cFos expression in cells located at three different rostrocaudal levels of the PAG (−8.30, −7.7 and −7.1 mm relative to Bregma) in both hSYN-ChR2-eYFP and control rats following the repeated photostimulation protocol. We used both nNOS and NeuN expression to identify the dorsomedial, dorsolateral, lateral and ventrolateral PAG subdivisions, according to the Paxinos and Watson (Paxinos and Watson, 2005a) rat brain atlas and nNOS staining (Bandler and Shipley, 1994; Chong et al., 2019).

In order to assess the regions that were potentially activated by PAG stimulation, we compared cFos + cells in different respiratory structures between hSYN-ChR2-eYFP and naïve rats following the photostimulation protocol. Within the PBN, we assessed the cFos + cell expression in the lateral PBN (−8.9, −9.2 and −9.5 mm relative to the Bregma), in the medial PBN (−9.5 and −9.8 mm relative to Bregma) and in the Kölliker Fuse nucleus (−8.8 and 9.2 mm relative to Bregma) according to the Paxinos and Watson (Paxinos and Watson, 2005a) rat brain atlas. We also assessed the cFos expression in the LC (−9.2, −9.5, −9.8 and −10.1 mm relative to the Bregma), in the caudal NTS (from 300 μm rostral to 900 μm caudal to the opening of the central canal) and along the ventral medulla, medial to the ventral extension of the spinal trigeminal tract below the nucleus ambiguus (750 × 750 μm box) to encompass the subdivisions of the ventral respiratory column (from 900 μm rostral of the caudal tip of the facial nucleus to the caudal tip of the lateral reticular nucleus). We also analyzed the expression of cFos in the intermediate reticular formation (iRT) dorsal to the nucleus ambiguus, in the region inclusive of the post-inspiratory complex, PiCo (500 × 750 μm portrait box). Because no additional phenotyping of neurons was performed (only cFos, GFP and NeuN expression was assessed) we couldn’t make strong conclusions regarding the functional significance of the histological findings. Data are illustrated as average cell counts ± SD, and comparison were performed between ipsi and contralateral sides (paired T-test) to the injections and between control and hSYN-ChR2-eYFP (unpaired T-test).

### EMG signal analysis

Raw EMG traces were collected from the Diaphragm (DIA), Genioglossus (GG) and Abdominal muscles (ABD) using LabChart8 (AD Instruments) and the absolute values were rectified and filtered with a time constant of 0.2 ms to yield an integrated trace (∫EMG). Instantaneous respiratory rate was extracted from ∫EMG_DIA_ signal as the time between consecutive peaks of DIA activity. All respiratory signals and the laser output were then exported to MATLAB (MathWorks) for further analysis using custom scripts. Figures were plotted in MATLAB and edited using CorelDraw (V16, Corel Corporation, Ottawa, Canada).

Muscle activity following bulk stimulation with 10 s pulses or high-frequency trains was analysed in two ways. The relative change in ∫DIA_EMG_ and ∫GG_EMG_ activity was compared to baseline by normalising the mean peak amplitudes during the stimulation to the mean peak amplitude of an equivalent (10 s) period of baseline activity preceding stimulation. Given ∫ABD_EMG_ does not typically show consistent oscillations under baseline conditions, the mean ∫ABD_EMG_ amplitude across the full duration of the stimulus burst was normalized to the mean of the baseline period preceding stimulation. The same procedure was done to analyse changes in respiratory rate during bulk stimulation. A grand mean was then computed across all baseline-stimulus burst pairs for each area and stimulus type.

Analysis of the respiratory response following brief (250 ms) laser pulses depends critically on the definition of the beginning and ending of the respiratory cycle. Triggers on the beginning of the cycle were calculated as the inflection points in the integrated signal (local peaks in the second derivative of ∫DIA_EMG_ signals or ∫GG_EMG_ for genioglossal muscle analysis) where the first derivative of integrated signals were positive. Peaks in the second derivative as well as the peak amplitudes of each cycle were extracted using the *findpeaks* function in MATLAB. Any identified peaks associated with noise were removed using a custom algorithm to systematically check that, 1) the number of cycles was equal to the number of identified peaks, and that 2) a single peak always proceeded from a single identified cycle start point. Once suitable triggers and peaks had been defined, cycles were split into “stimulus” cycles during which a laser pulse occurred, and “natural” cycles without any interfering laser stimuli. All time points in a cycle could therefore be converted to units of phase (degrees) by dividing the time elapsed since the beginning of the cycle by the mean cycle duration of all “natural” cycles and multiplying by 360.

Initial analyses were done by aligning 4 s segments of each ∫EMG around each laser pulse and averaging across stimuli to produce a stimulus-triggered average. Moreover, the 4 s timebase could be converted to units of phase as described above and segregated into phase bins spanning 400° before and after the stimulus. Binning the STA in this manner allowed us to average across all experiments to compare the elicited muscle activity at similar points in the respiratory cycle.

Finer analysis of the respiratory reset was done by calculating the phase at which the stimulus arrived, termed the “stimulus phase”, and the ‘induced phase” delay at which the next cycle began, as per previous studies (Pagliardini et al., 2011; Alsahafi et a., 2015) (Figure 3D). When reset does not occur, the induced phase and the stimulus phase add up to 360°, forming a linear relationship when plotted with respect to each other. Deviation from this linear relationship indicates an altered timing of the cycle proceeding from laser stimulation (Pagliardini et al., 2011; Alsahafi et al., 2015). Further analysis of the amplitude and duration of the response following reset stimulation was done by normalizing the evoked ∫EMG peak amplitude and cycle duration to their corresponding measures during natural breathing across the full range of stimulus phases. Thus, if brief stimulation did not produce any systematic changes to the peak activity or duration of EMG bursts this analysis would yield a constant value near 1 as a function of stimulus phase.

### Exclusion Criteria

Simultaneous recordings of EMG activity in the diaphragm, genioglossal, and abdominal muscle were analysed for signal quality on an ongoing basis throughout the experiment based on the relative background noise, clear interference between the recording channels, strong contamination of the EMG with heartbeat related artifacts, and the presence of clear electrical artifacts. Poor signal quality in any of these regards for any one of the three EMG signal during periods of baseline or stimulation was noted and that signal was removed from further analyses. Furthermore, experiments in which the brief pulse protocol did not adequately sample the full 360-degree range of stimulus phases were similarly removed from the results of the reset response fitting (see Results, Figure 4G).

Our experiments investigated several variables including stimulation in differing regions, with differing stimulation protocols, with a unilateral or bilateral approach, and in some cases with different viral vectors. Moreover, these experiments were conducted within-subjects as much as possible except between groups of different viral vectors, or between unilateral versus bilateral stimulation. In these cases, analyses were first conducted separately and pooled only when no statistical differences were noted (see Results). As a result of the exclusion criteria above and the within-subjects design, the number of replicates differ significantly for a given experimental condition compared to the total number of animals noted above. The number of replicates which remained for a specific analysis are noted in the relevant result sections below.

### Statistical methods

Relative ∫EMG amplitudes and relative respiratory rates were compared for statistical significance above the baseline value of 1 using one-tailed statistical tests. Either a one-sample *t*-test, signed test or Wilcoxon signed-rank test were conducted based on the normality and symmetry of the given distribution as assessed via a Shapiro-Wilk’s test of normality. Further comparison of relative measures across brain areas was performed by either a Kruskal–Wallis test together with a Dunn’s test for multiple post-hoc comparisons or by a 1-way ANOVA with the Tukey’s Honestly Significant Difference post-hoc testing procedure as appropriate depending on the normality of the underlying distributions as determined by a Shapiro-Wilk’s test. The results of unilateral and bilateral photostimulation were combined in the reset analyses as they did not result in any significant differences. Similarly, the data following AAV 2/5 and AAV 9 viral injections were combined in all analyses as they did not result in significantly different effects (see Results section).

## Results

Figure 1 shows a summary of the locations of GFP expression 4 weeks following injection of hSYN-ChR2-eYFP viral particles at the level of PAG as well as the location of the fiber-optic tracts (n = 17). Because the GFP expression was filling preferentially neuronal processes rather than the soma of infected cells, we did not perform cell counting of GFP + neurons but assessed the intensity of fluorescence in the brain tissue. By using double-labelling with an anti-nNOS antibody which delineates the dorsolateral PAG (Figure 1B) and the dorsal raphe structures (Bandler and Shipley, 1994; Chong et al., 2019), GFP expression was confirmed to be intensely localized to the lateral and ventrolateral PAG with limited expression in the more dorsal and ventral portions of the PAG. The most intense GFP labelling was localized between −8.30 and −7.30 mm from bregma, according to a rat brain atlas (Paxinos and Watson, 2005b). To further determine the area of activation in optogenetic experiments, we compared the number of cFos + cells within the PAG subdivision in hSYN-CHR2-EYFP rats with control rats (n = 3) that underwent the same PAG photostimulation protocol (without showing any physiological response). In control rats, a modest number of cFos + cells were observed in each PAG subdivision, with the highest number being in the lateral PAG and ventrolateral PAG (IPSI: dmPAG: 8.4 ± 3.1 cFos + cells/section; dlPAG: 17.3 ± 6.6 cFos + cells/section; lPAG: 30.6 ± 16.4 cFos + cells/section; vl PAG: 39.8 ± 21 cFos + cells/section). No difference was observed between the ipsilateral and contralateral sides to the photostimulation in control rats (CONTRA: dmPAG: 6.7 ± 1.4, cFos + cells/section, p = 0.21; dlPAG: 17.1 ± 6.8 cFos + cells/section, p = 0.8; lPAG: 31.2 ± 9.8 cFos + cells/section, p = 0.88; vlPAG: 49.3 ± 12.2 cFos + cells/section, p = 0.37). In hSYN-CHR2-EYFP rats, an increased number of cFos + cells was observed in each subdivision of the ipsilateral PAG compared to control rats, with the exception of the dmPAG (dmPAG: 19.3 ± 6.5 cFos + cells/section, p = 0.06; dlPAG: 53.7 ± 19.9 cFos + cells/section,p = 0.04; lPAG: 88.9 ± 24.5 cFos + cells/section, p = 0.03; vlPAG: 141.4 ± 1.2 cFos + cells/section; p = 0.02). Furthermore, cFos + cells were more numerous ipsilateral to the injection side and the optic fiber tract compared to the contralateral side in the lateral PAG (CONTRA: dmPAG: 19.2 ± 2.7 cFos + cells/section, +0.4%; p = 0.97; dlPAG: 34.7 ± 12.6 cFos + cells/section, p = 0.05; lPAG: 40.5 ± 10.6 cFos + cells/section, p = 0.03; vlPAG: 89.6 ± 17.1 cFos + cells/section, p = 0.16). These results suggest that the observed respiratory function changes described below primarily stem from the activation of the ipsilateral (infected) lateral and ventrolateral PAG which may also indirectly activate the adjacent subdivisions and the contralateral side through local networks.

### PAG stimulation potently stimulates respiratory EMG activity and elevates breathing rate

**FIGURE 2:**
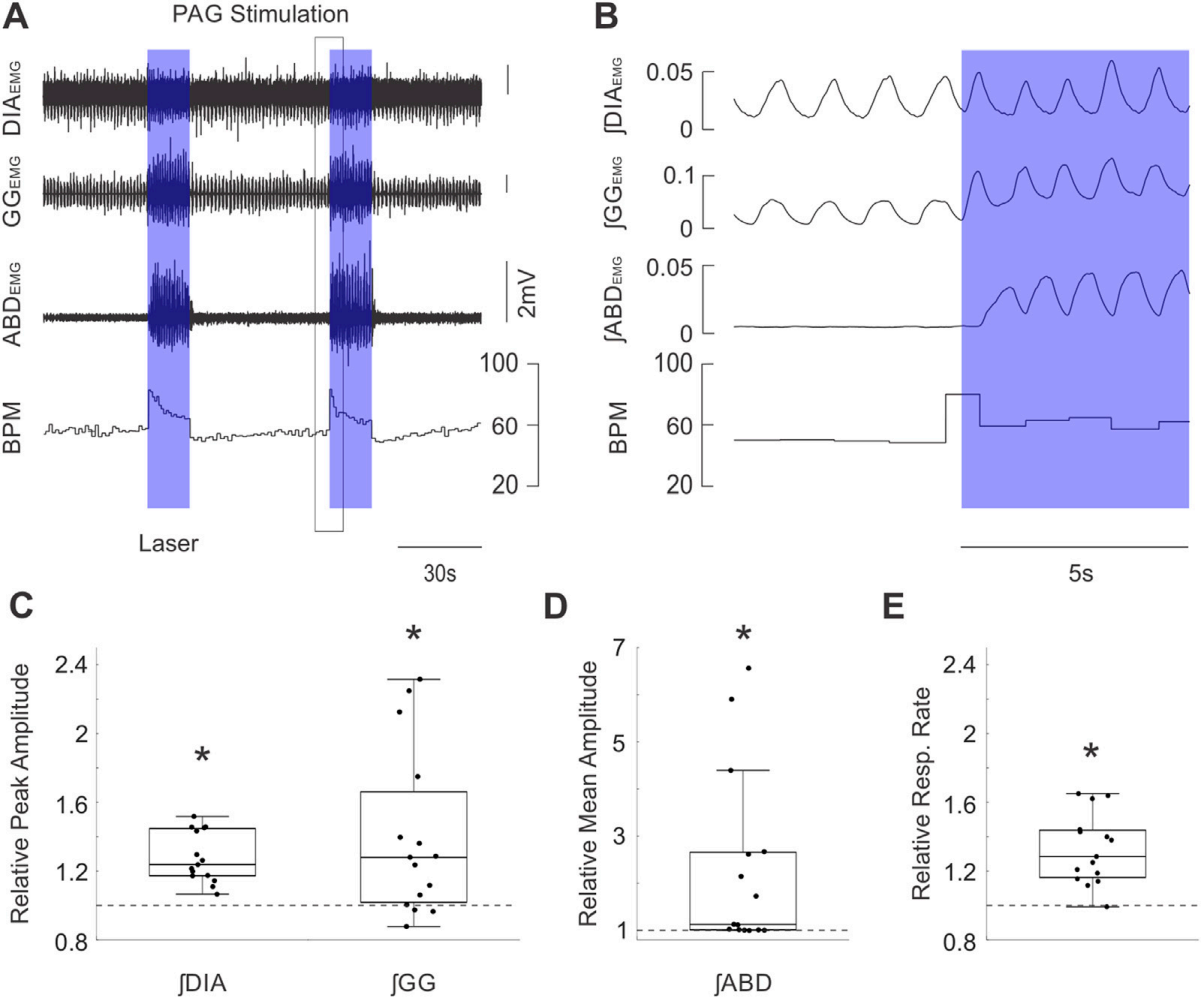
Optogenetic activation of PAG potently stimulates inspiratory and expiratory muscle activity and increases respiratory rate. **(A)** Example traces of raw diaphragm (DIA), genioglossus (GG), and abdominal (ABD) _EMG_ activity together with breaths per minute (BPM) before and after 2 bursts of high frequency photostimulation (blue boxes). Photostimulation potently increases EMG activity at all three sites while also dramatically increasing BPM. Black box indicates the zoomed area shown in **(B)**. **(B)** Expanded integrated traces of EMG activity from A before and after the onset of laser stimulation (blue box) showing the rapid enhancement of EMG activities along with increasing respiratory rate. **(C)** Peak amplitude of integrated DIA_EMG_ and GG_EMG_ activity during laser stimulation normalized to activity before stimulation (n = 15). Dots correspond to individual animals. Boxes define the 2^nd^ and 3^rd^ quartiles surrounding the median (solid line), whiskers show 1^st^ and 4^th^ quartiles. Dashed lines at 1 indicate the amplitude of baseline activity, stars mark significant differences from 1 as measured by a sign test or *t*-test at the p = 0.05 level. **(D)** Similar to C, dots indicate the mean amplitude of integrated ABD_EMG_ activity relative to baseline activity (dashed line) for each animal. **(E)** Similar to C &D, dots indicate the mean respiratory rate during stimulation of PAG relative to baseline rate (dashed line).

In our optogenetic experiments, the first aim was to determine how respiratory musculature responded to spatially and temporally controlled stimulation of the PAG. As demonstrated in the sample recordings of Figure 2A (zoomed view in Figure 2B), optogenetic stimulation with high-frequency trains of laser pulses elicited strong activation of the ABD and GG _EMG_ activity which lasted for the full duration of the stimulus before returning to baseline levels of activity at the end of the stimulus. High-frequency stimulation also elicited a subtler increase in ∫DIA_EMG_ activity and induced an immediate augmentation in respiratory rate (Figures 2A,B). Quantification of these effects across all rats (n = 15) indicated that our stimulation significantly elevated peak ∫DIA_EMG_ (median = 1.24, *t* (15) = 7.38, *p* < 0.001), ∫GG_EMG_ (median = 1.28, *S* = 12, *p* = 0.035), and respiratory rate (median = 1.29, *t* (15) = 6.21, *p* < 0.001) relative to baseline respiration (Figures 2C,E). Moreover, we observed a wide distribution of expiratory modulated ∫ABD_EMG_ activation, ranging from 1.0 to ∼6.5-fold increases relative to baseline with a median of ∼1.1 times baseline activity (*S* = 15, *p* < 0.001) (Figure 2D). Stimulation of the PAG with 10 s continuous pulses of laser light produced results which qualitatively and quantitatively matched the results described for stimulation with high-frequency pulse trains.

### Brief pulse stimulation of PAG resets the respiratory rhythm and alters EMG activity in either a phase-dependent or phase-independent manner

**FIGURE 3:**
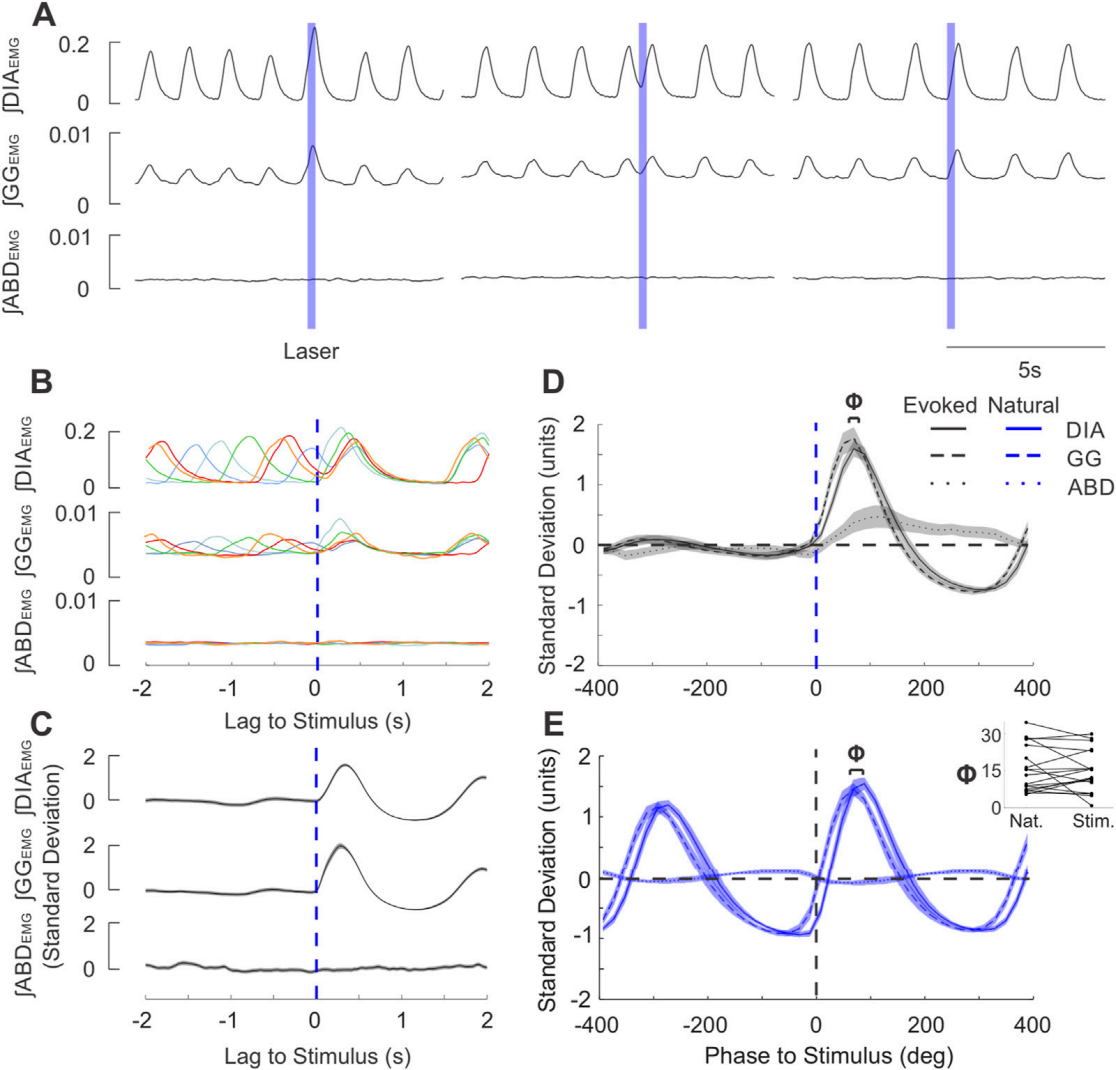
Brief stimulation of the PAG elicits phase-locked EMG activity after a short delay in the same sequence as natural breathing. **(A)** Example integrated EMG traces for three different instances of brief (250 ms) pulse stimulation (blue boxes) delivered at different phases of the natural ongoing breathing rhythm. Note the increased DIA and GG amplitude and duration in the left trace and the rapidly-evoked activity shortly after laser onset in the middle and rightmost traces. **(B)** Stimulus-triggered overlay of integrated EMG activity. Five breathing cycles during which stimulus pulses arrived at different phases of the ongoing respiratory cycle are overlaid and time-locked to the onset of laser pulses (lag = 0s). Apparent reset occurs at short latencies and is also observed for the subsequent cycle. **(C)** Stimulus-triggered average across all cycles for the experiment shown in **(A)**, **(B)**. Integrated traces were z-scored to highlight the prominent stimulus-evoked activity. Shaded areas around the mean traces indicate ± S.E.M. **(D)** Mean stimulus-triggered activity for all brief PAG stimulation experiments (n = 26). Stimulus-triggered averages from each experiment were binned into phases of the cycle according to the length of their respective natural breathing cycle, ranging from 400° before the stimulus (−400 deg) to 400° after the stimulus (400 deg)., The amplitude of traces were normalized by the standard deviation of spontaneous baseline traces prior to averaging. Grouped-average stimulus-triggered activity from DIA (continuous line), GG (dashed line), and ABD (dotted line) are overlaid together with the shaded areas indicating ± S.E.M. Blue dashed line indicates the onset of stimulus. **(E)** Diaphragm-triggered averages of integrated EMG traces as recorded baseline recordings for the experiments shown in D (n = 26). Activity was normalized in both phase and amplitude measures similary to that described in D and then averaged across all experiments. Phase lag of 0 deg occurs at the onset of diaphragmatic EMG bursts. Above the traces, Φ indicates the average phase lag between the onset of GG (dashed) and DIA (solid) EMG activity. Inset: GG-DIA phase lag (Φ in **(D, E)** for natural breaths and stimulus-evoked activity (n = 18).

Exploiting the temporal precision of optogenetic stimulation, we further investigated whether brief (250 ms) pulses of light in the PAG were sufficient to alter the timing of respiratory muscle activities. Indeed, we consistently observed a stimulus-locked response in peak ∫DIA_EMG_ and ∫GG_EMG_ activity following brief stimulus pulses (Figure 3A). Effectively, this stimulation altered and often advanced the timing of the subsequent inspiratory EMG activation within the existing respiratory cycle to reset the ongoing rhythm itself (Figure 3B). This reset was particularly evident in the consistent stimulus-locked occurrence of inspiratory EMG activity at short (12–125 ms) latencies following stimulation during the expiratory period and could be observed with individual overlaid traces (Figure 3B), as well as in the stimulus-locked averages (Figure 3C). Indeed, the absence of oscillatory signatures in the pre-stimulus averaged trace in Figure 3C and the consistent stimulus-locking in the overlaid selected traces in Figure 3B indicates that pulses elicited stimulus-locked inspiratory muscular activity at all phases of the respiratory cycle.

By normalising traces in the time domain to the average respiratory cycle, we characterized the relative phasing and pattern of activation of respiratory muscles evoked by PAG photostimulation across all experiments (n = 26). Short pulses of light evoked EMG activation which began with the genioglossal muscle, followed by an increase in diaphragm activity and ending with a slow and smaller enhancement in abdominal activation (Figure 3D). This pattern of muscle recruitment was essentially identical for spontaneously-occurring respiratory activities triggered on diaphragmatic activity; except for the abdominal activation, which was absent (n = 18, Figure 3E). Indeed, both the delay (Φ in terms of cycle phase, Figure 3E inset, time delay not shown) between the peak genioglossal and diaphragmatic burst for stimulus evoked activity and spontaneous breathing were not significantly different (Φ: *t* (17) = 0.562, *p* = 0.582, Figure 3E inset; time delay: *z* = 0.152, *p* = 0.879). Thus, short duration stimulation of the PAG was capable of evoking prominent stimulus-locked EMG activity changes which did not differ from the typical pattern of inspiratory muscle activation observed under natural (spontaneously-occurring) conditions.

**FIGURE 4:**
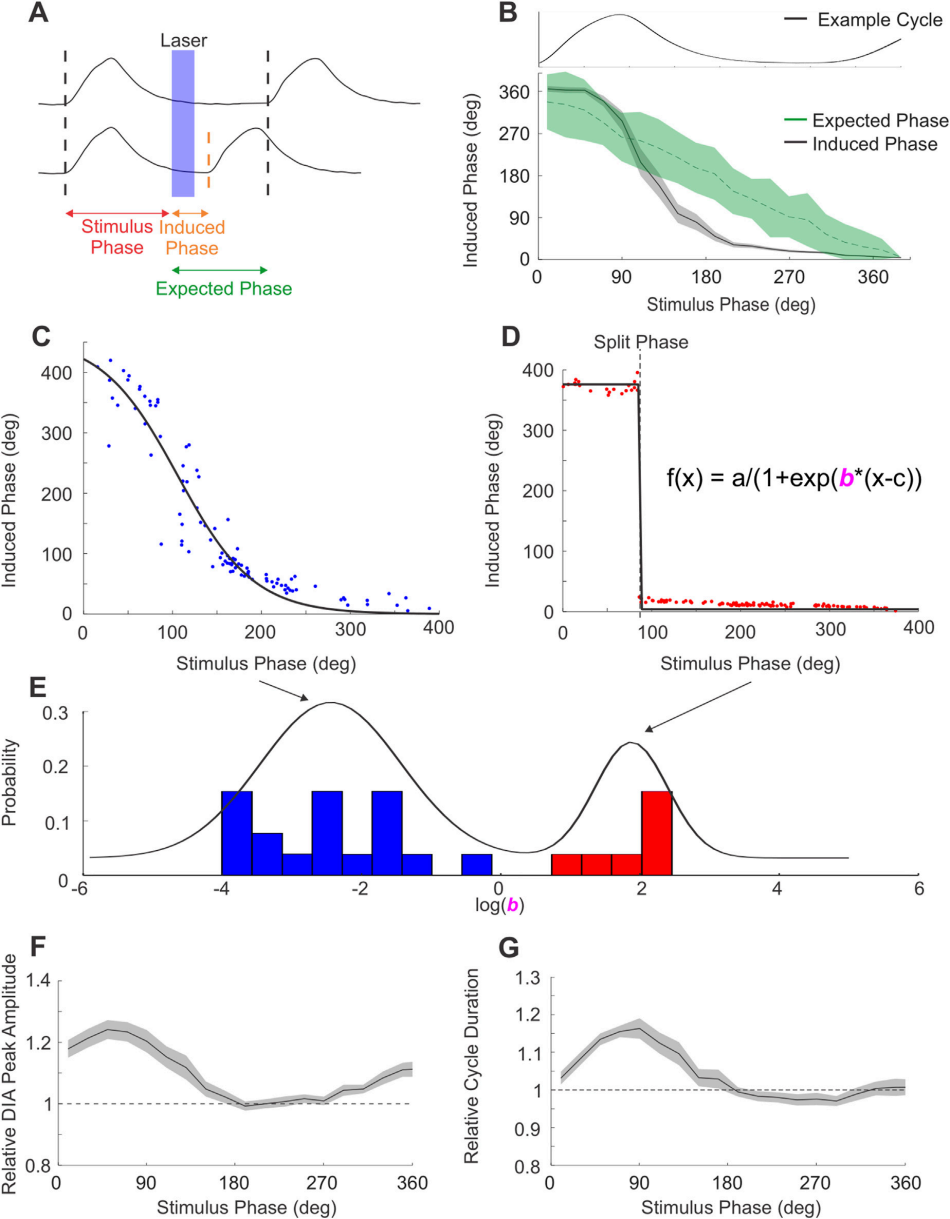
PAG stimulation produces 2 types of respiratory reset. **(A)** Diagram of phase measures obtained from integrated DIA_EMG_ traces used to quantify respiratory reset. Top trace exemplifies the expected phase of the cycle subsequent to photostimulation if stimuli did not elicit any changes to respiratory timing. Bottom trace shows the induced phase between stimulus and an advanced bout of EMG activity elicited by the laser pulse (in blue). Differences between the expected and induced phase indicate changes in respiratory timing. **(B)** Stimuli arriving to different phases of the respiratory cycle (example respiratory cycle aligned and overlaid at the top of the panel) altered the onset of the subsequent cycle in a phase-dependent manner. Induced phase for DIA activity across all experiments (n = 26, black), shaded area represents ± S.E.M. Expected phase (green) indicates the mean and 95% confidence limits derived from a spontaneously-derived surrogate dataset. Stimulation can be seen to significantly alter the induced from the expected phase of inspiration at multiple phase points of the ongoing rhythm. **(C)** Induced phase curve for an example experiment following phase-dependent reset. Dots indicate individual stimulus pulses, black line indicates a sigmoid fit. **(D)** Similar to C, induced phase curve for an experiment exemplifying phase-independent reset. Sigmoid fits in C,D are modeled using equation in D, by modifying the slope parameter “b”. **(E)** Distribution of slope parameter for all PAG-stimulation experiments (n = 25) in log space. Mixed Gaussian with 2 components overlaid in black, highlighting the separation between the phase-dependent (blue) and phase-independent (red) reset. **(F)** Peak integrated DIA_EMG_ activity evoked by stimulus pulses arriving at different phases of the respiratory cycle relative to the mean peak activity of breaths during baseline (dashed line at 1). Data represent mean of all PAG-stimulation experiments (n = 26), shaded area represents ± S.E.M. **(G)** Similar to F, duration of the respiratory cycle evoked by PAG stimulation as a function of stimulus phase, relative to the average duration of natural cycles (dashed line at 1).

We next examined whether the respiratory cycle was reset in a phase-independent manner. To this end, we computed the phase of the respiratory cycle at which stimuli were delivered, as well as the phase of the subsequent (induced) cycle (Figure 4A c. f. (Pagliardini et al., 2011; Alsahafi et al., 2015)). These were compared to data collected during the absence of photostimulation by randomly assigning the same set of stimulus phases to traces of ongoing spontaneous respiratory cycles. Our summarized results for ∫DIA_EMG_, shown in Figure 4B (n = 26), indicate that the average induced phase during photostimulation clearly deviates from the expected linear relationship with stimulus phase observed in its absence. Instead, the evoked change in inspiratory timing was dependent on the phase of the cycle at which the stimulus was delivered, varying in a sigmoid relationship with an extended tail that covered the 0–360° range (Figure 4B). Stimuli arriving during inspiration (i.e., before or at the peak of ∫DIA_EMG_ activity) caused a consistent increased latency to the subsequent diaphragmatic burst that was reflected by an increased induced phase above the expected phase (i.e. at, or slightly greater than, 360°). Conversely, stimuli arriving during the expiratory phase (i.e., after the peak of the natural activation of ∫DIA_EMG_ activity) caused an advance in the timing of the proceeding cycle which resulted in a relatively consistent induced phase that was substantially below the expected phase (Figures 4B,C). Interestingly, in 28% of experiments (7/25), the induced phase varied as a step-function, immediately transitioning from a plateau (>360°) during inspiratory phases to a small (<20 deg) phase delay during expiratory phases as shown in Figure 4D. This reflects dramatic and phase-independent reset at all points of the respiratory cycle. However, for most experiments, the shape of the induced phase curve was fit well by a sigmoid function (Figure 4C) which suggested a more phase-dependent form of reset, especially during the ongoing inspiratory phase. The slope parameter (‘b”) of this sigmoidal fit was found to be bimodally distributed in our data, suggesting the existence of 2 distinct types of reset in our recorded population (Figure 4E). Despite these differences, we note that in all cases, stimulus pulses did indeed reset the ongoing respiratory cycle as induced phase was substantially below the expected phase during the expiratory period (Figures 4B–D).

For stimulus trials in which the photostimulation was delivered just after initiation of diaphragmatic EMG activity, our triggering analysis was unable to accurately identify whether stimulation evoked a further burst of EMG activity which could be differentiated from the ongoing DIA activity already present. In these cases, we often observed (as shown in Figure 3A, left-most panel) an enhanced peak activation of both the ʃDIA and ʃGG _EMG_ signals. This suggested that respiratory reset was likely occurring during the ongoing inspiratory phase of the respiratory cycle as well. This was also consistent with the robust stimulus-locked averages in the absence of any oscillatory activity pre-stimulus previously shown (Figure 3D) and the consistent ≥360° phase shift of the next inspiratory cycle occurring when photostimulation was delivered during the inspiratory phase of respiration (Figure 4B).

In order to more accurately characterize this effect, we analysed how elicited EMG activity differed as a function of stimulus phase by comparing the relative peak amplitude and duration of respiratory cycles evoked by stimulation to those recorded during natural breathing. As can be seen in Figure 4F, the peak amplitude of ʃDIA _EMG_ activity was significantly increased compared to natural breathing by a factor of ∼1.2 when stimulus pulses were delivered in the 0–100-degree phase period, which was coincident with spontaneous inspiratory muscle activation. Indeed, the duration of the inspiratory phase of respiratory cycles evoked by photostimulation during this period was also increased by a factor of ∼1.2 (Figure 4G). This period of EMG facilitation coincided well with the increase in measures of induced phase occurring during inspiration as shown in Figure 4B (i.e., ≥360°). Photostimulation delivered outside of this window did not substantially increase the amplitude nor the duration of the induced EMG activation of either the diaphragm or genioglossus (latter not shown) musculature. In all cases, the analyses above, when applied to ∫GG_EMG_, yielded results which closely matched those described above for ∫DIA_EMG_.

### Repeated stimulation of PAG increases cFos expression in neurons of the ventral respiratory column, the intermediate reticular formation and the locus coeruleus

To further explore the effect of PAG photostimulation on the respiratory network, we analyzed the pattern of cFos expression in both hSYN-ChR2-eYFP (n = 4) and control rats (n = 3) following a high-intensity, repeated photostimulation protocol targeting the PAG. We specifically counted the average number of cFos + cells in regions that are well known to receive projections from the PAG and that they could contribute to the effects described in our study. We focused our investigation on the PBN (lateral and medial parabrachial and Kölliker fuse nuclei) the LC, the NTS, and the reticular formation above the nucleus ambiguus (encompassing the area of the post-inspiratory complex, PiCo, upper airway premotor neurons and other neurons involved in orofacial behaviours) (Trevizan-Baú et al., 2021b; Farkas et al., 1997; MacDonald et al., 2024; Lubejko et al., 2024; Huff et al., 2023; Toor et al., 2019; McElvain et al., 2018), as well as the various subdivisions of the ventral respiratory column ipsilateral to the side of infection and photostimulation (Trevizan-Baú et al., 2021b; Del Negro et al., 2018).

**FIGURE 5:**
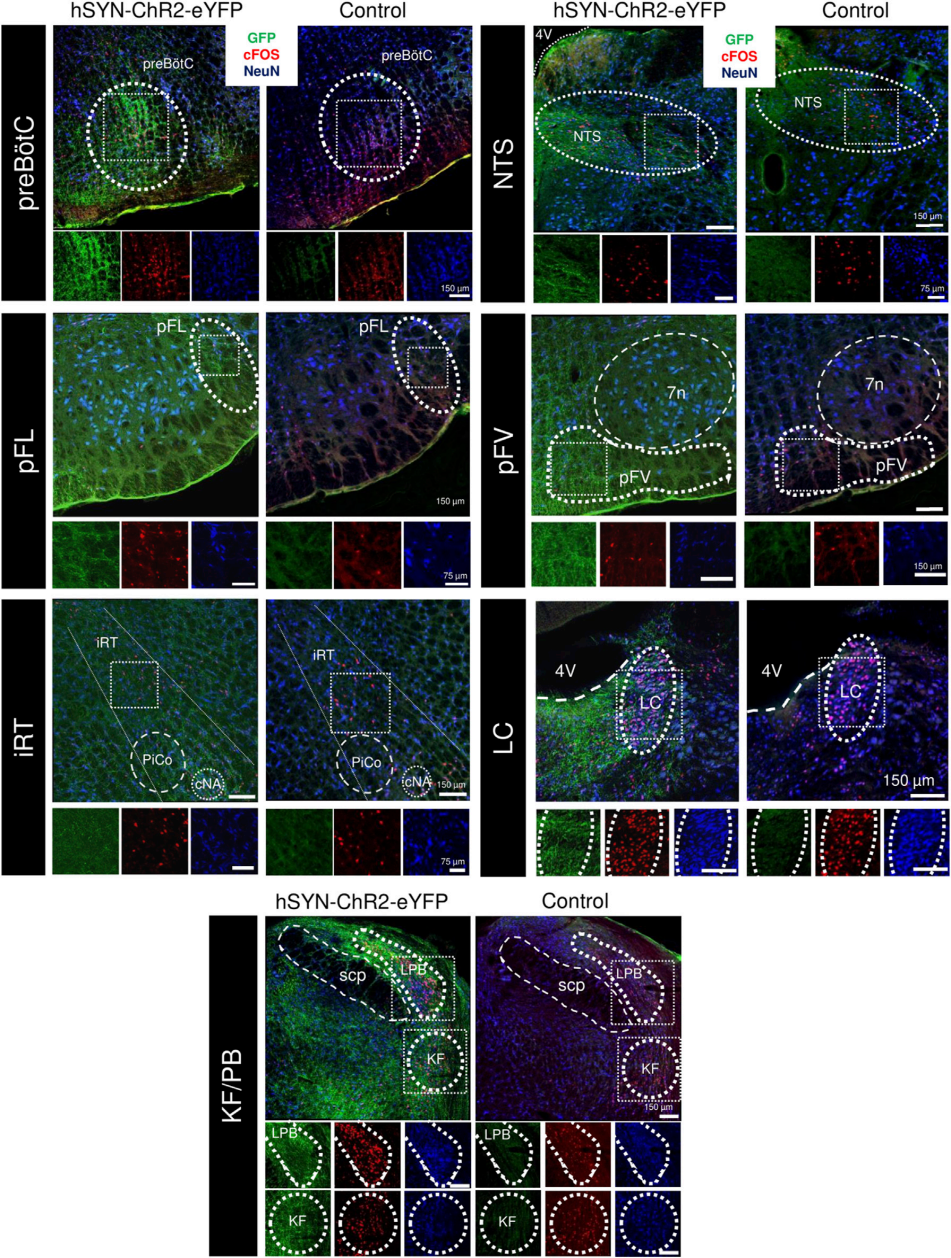
cFOS expression following repeated PAG stimulation in the respiratory network. Overlaid immunofluorescence for GFP (green), cFOS (red), and NeuN (blue) in rats infected with the hSYN-ChR2-eYFP virus (left) and a naive animal (right) in the preBötzinger Complex (preBötC, A), nucleus of the solitary tract (NTS, B), the lateral parafacial (pFL, C), the ventral parafacial (pFV, D), the intermediate reticular formation (iRT, E) the locus coeruleus (LC, F) and the parabrachial nuclei of the Kölliker-Fuse (KF) and lateral parabrachial (LPB, G). Insets below show high power magnification of immunostained tissue in separate channels. 7n, Facial motor nucleus; cNA, nucleus ambiguus, pars compacta; PiCO, post-inspiratory complex; 4V, fourth ventricle; scp, superior cerebellar peduncle.

Within the PBN, no significant difference was observed in the number of cFos + cells in either the lateral parabrachial (control: 108.9 ± 10.8; hSYN-ChR2-eYFP: 114.8 ± 29.8 cfos + cells/section; p = 0.76), the medial parabrachial (control: 5.6 ± 1.9; hSYN-ChR2-eYFP: 8.3 ± 4.3 cfos + cells/section; p = 0.4) or the Kölliker Fuse nuclei (control: 65.5 ± 16.8; hSYN-ChR2-eYFP: 62.4 ± 1.7 cfos + cells/section; p = 0.76) (Figure 5). While the number of cFos + cells was higher in hSYN-ChR2-eYFP compared to control rats in the LC (37.8 ± 3.6 vs. 17.8 ± 7.4, p = 0.01) as well as the intermediate reticular formation (5.6 ± 2.2 vs. 15.1 ± 4.2, p = 0.03), the increase did not reach significance in the caudal NTS (80.0 ± 10.2 vs. 48.8 ± 18.2; p = 0.06) (Figure 5).

We then investigated cFos + expression along the ventral medulla, in between the spinal trigeminal tract and the inferior olive, and below the nucleus ambiguus between control and hSYN-ChR2-eYFP rats (Figure 5). Overall, the number of cFos + cells was increased through the rostrocaudal extension of the respiratory column in hSYN-ChR2-eYFP (600.6 ± 50.52 cells/rat) compared to control rats (314.8 ± 10; p = 0.0006). When we compared the cFos expression in the different subregions of the respiratory columns in the ventral medulla, we observed an increase in cFos expression lateral to the facial nucleus, in the region corresponding to the location of the pFL (Pisanski et al., 2024) (pFL, control: 5.8 ± 2.1; hSYN-ChR2-eYFP: 19.0 ± 6.7 cfos + cells/section; p = 0.03), caudal to the facial nucleus, in the region containing both Bötzinger Complex neurons and C1 cells (control: 24.5 ± 3.8; hSYN-ChR2-eYFP: 44.67 ± 3.7 cfos + cells/section; p = 0.003), and the preBötC (control: 31.7 ± 6.5; hSYN-ChR2-eYFP: 63.01 ± 10.1 cfos + cells/section; p = 0.01). Surprisingly, no difference was observed in either the ventral parafacial region, which includes the retrotrapezoid nucleus (pFV, control: 6.33 ± 1.89; hSYN-ChR2-eYFP: 9.8 ± 5.9 cfos + cells/section; p = 0.4), the rostral VRG (naïve: 37.4 ± 21.9; hSYN-ChR2-eYFP: 53.7 ± 15.3 cfos + cells/section; p = 0.35) or the caudal VRG (naïve: 23.86 ± 7.3; hSYN-ChR2-eYFP: 28.5 ± 6.14 cfos + cells/section; p = 0.45). These results suggest that repeated stimulation of PAG significantly increases the number of activated cFos + cells in the LC, the intermediate reticular formation, pFL, BötC and preBötC.

### Photostimulation of PAG synaptic terminals in preBötC and pFL does not entirely replicate the complete effects of direct midbrain stimulation

Our next aim was to investigate how the effects of PAG stimulation might be mediated by stimulating the terminals of its downstream projections to critical respiratory rhythmogenic areas of the preBötC and the pFL, where we also observed increased expression of cFos + cells in our histological analysis. To aid in this endeavour, we conducted experiments using the same optogenetic virus used in the previous experiments (hSYN-ChR2-eYFP, AAV2/5 serotype; n = 4, 3 respectively). We also tested the response using the same construct (hSYN-ChR2-eYFP) in an AAV9 serotype (n = 4 for both areas), since it has been shown to be able to preferentially localize opsins in terminals (Jackman et al., 2014).

In most cases, our results remained consistent between both viral serotypes as we did not find any significant differences between them following a 20 Hz pulse train stimulation protocol directly into PAG (∫DIA_EMG_: *t* (13) = −0.291, *p* = 0.776; ∫GG_EMG_: *t* (13) = −1.832, *p* = 0.089; ∫ABD_EMG_: *U* = 18, *p* = 0.078; Resp. Rate: *t* (13) = −0.595, *p* = 0.562), for nearly all measures following stimulation in the preBötC (∫DIA_EMG_: *t* (6) = −0.09, *p* = 0.932; ∫GG_EMG_: *t* (6) = −0.05, *p* = 0.964; Resp. Rate: *t* (6) = 0.519, *p* = 0.623), nor in any measures following stimulation in the pFL (∫DIA_EMG_: *U* = 16, *p* > 0.999; ∫GG_EMG_: *U* = 16, *p* > 0.999; ∫ABD_EMG_: *t* (5) = 1.213, *p* = 0.279; Resp. Rate: *t* (5) = 0.723, *p* = 0.502). Significant differences in ∫ABD_EMG_ activity were only found when stimulating in the preBötC when using AAV2/5 relative to AAV9 (*t* (6) = 3.165, *p* = 0.019). However, given the small median difference in the AAV2/5 group relative to baseline breathing (0.994 times baseline) and the negligible effect size between types of viral injection (*d* = 0.0078), we consider this as a spurious effect. Thus, all results reported below for all areas are pooled between viral types.

#### preBötC stimulation

**FIGURE 6:**
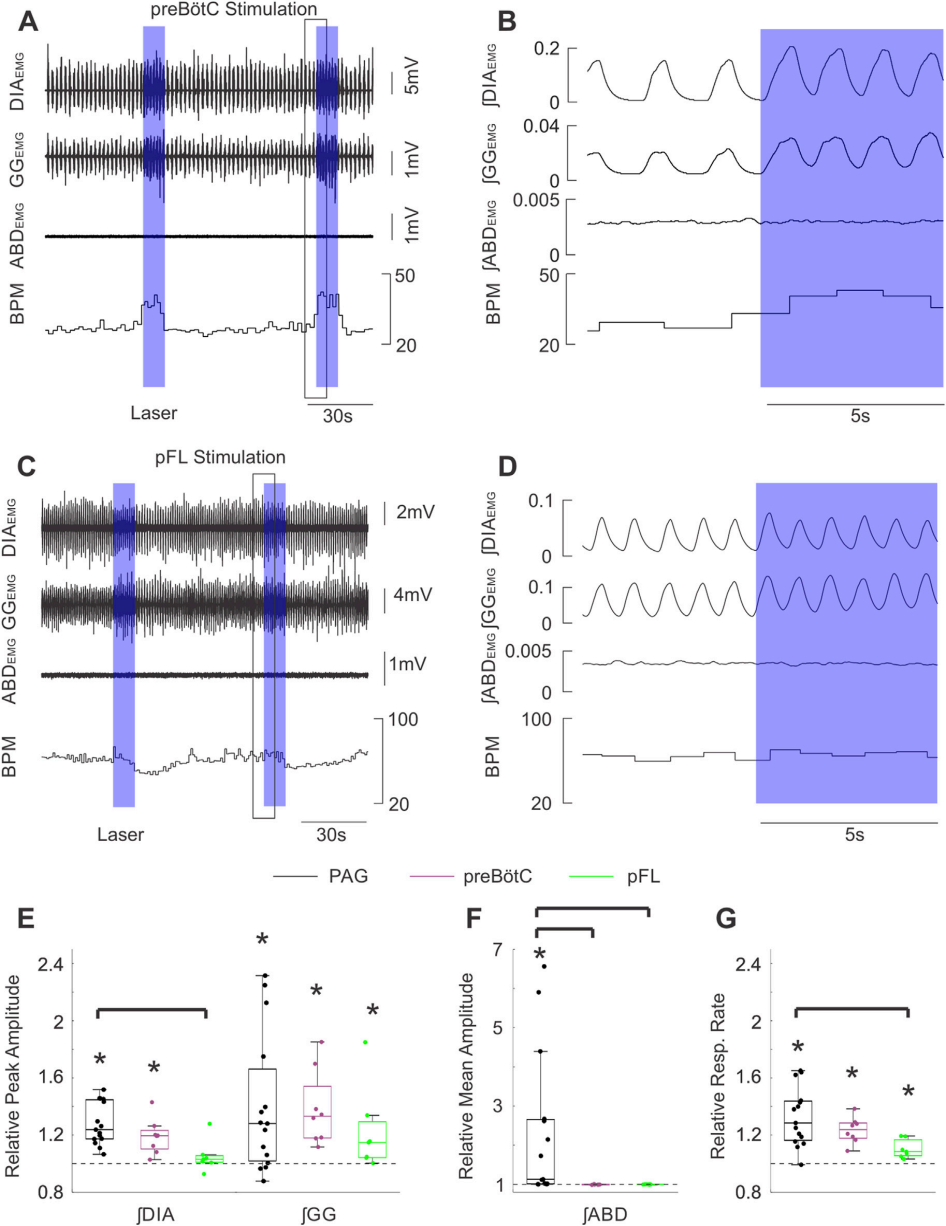
Stimulation of PAG terminals in the preBӧtC and pFL does not produce the same effects as direct PAG stimulation. **(A)** Example traces of raw diaphragm (DIA), genioglossus (GG), and abdominal (ABD) EMG activity together with breaths per minute (BPM) before and after 2 high frequency bursts of photostimulation of PAG terminals in preBӧtC (blue boxes). Photostimulation increases EMG activity in DIA and GG (but not ABD) sites and also increases BPM. Black box indicates the zoomed area shown in **(B)**. **(B)** Expanded integrated traces of EMG activity from A before and after the onset of laser stimulation (blue box) showing the enhancement of EMG activities in DIA and GG traces (although not ABD) along with and increased respiratory rate. **(C)** Similarly to A, example traces showing raw EMG activity and respiratory rate effects elicited by stimulating PAG terminals in pFL. **(D)** Magnified traces from the example in **(C)**. The effects of stimulation in pFL were more modest than that for the preBӧtC. **(E)** Peak amplitude of integrated DIA and GG _EMG_ activity following high frequency burst photostimulation of the PAG (black, n = 15), PAG terminals in preBӧtC (purple, n = 8), and PAG terminals in pFL (green, n = 7) relative to activity during baseline (dashed line at 1). Boxes indicate middle quartiles and median, whiskers denote outer quartiles. Stars indicate significance from baseline measures as determined by *t*-test. Brackets indicate a significant difference at p = 0.05 using one-way ANOVA with a post-hoc Tukey’s HSD test or Kruskal–Wallis with Dunn’s test post-hoc correction for multiple comparisons. **(F)** Similar to E, mean amplitude of integrated ABD _EMG_ activity during photostimulation relative to baseline activity (dashed line at 1). **(G)** Similar to E, mean respiratory rate during photostimulation relative to respiratory rate during baseline.

Stimulating PAG terminals in the preBötC bilaterally with high-frequency trains of laser light (Figure 6, n = 8) or 10 s pulses (not shown) elicited substantial increases in both ∫DIA_EMG_ and ∫GG_EMG_ activity, as well as increasing respiration rate (Figures 6A,B). Indeed, the peak of evoked diaphragmatic and genioglossal activity was found to be significantly elevated compared to baseline respiration (∫DIA_EMG_: *t* (7) = 4.38, *p* = 0.003; ∫GG_EMG_: *t* (7) = 4.16, *p* = 0.004) reaching a median of ∼1.2 and ∼1.3 times the peak of natural muscle activity respectively (Figure 6E). Relative respiration rate was also significantly increased by a factor of ∼1.2 (*t* (7) = 7.34, *p* < 0.001). Moreover, the increases in muscle activity and respiration rate following bilateral stimulation of the PAG fibres in the preBötC were found to be comparable on average to those elicited by direct stimulation of the PAG (∫DIA_EMG_: *F* (2,27) = 7.05, *p* = 0.003, Tukey HSD: *p* = 0.290; ∫GG_EMG_: *H* (2,27) = 1.87, *p* = 0.392; Resp. Rate: *F* (2,27) = 4.92, *p* = 0.015, Tukey HSD: *p* = 0.383) (Figures 6E,G).

On the other hand, unilateral stimulation of the PAG terminals (ipsilateral to the viral injection) in the preBötC (Supplementary Figure S1, n = 5) produced more modest effects on these respiratory measures. Integrated DIA_EMG_ activity was not significantly elevated (median = 1.05, *t* (4) = 2.48, *p* = 0.068), while ∫GG_EMG_ activity (median = 1.20, *t* (4), = 4.24, *p* = 0.013), and respiratory rate (median = 1.16, *t* (4) = 6.39, *p* = 0.003) were both significantly increased relative to baseline (Supplementary Figure S1C, E). Increases in ∫DIA_EMG_ activity (median = 1.05, *t* (4) = 2.48, *p* = 0.034), ∫GG_EMG_ activity (median = 1.20, *t* (4), = 4.24, *p* = 0.007), and respiratory rate (median = 1.16, *t* (4) = 6.3947, *p* = 0.002) relative to baseline (Supplementary Figure S1C, E). Moreover, this evoked increase in diaphragmatic activity was found to be significantly lower compared to the activity evoked via direct stimulation of the PAG (*F* (2,25) = 6.03, *p* = 0.007, Tukey HSD: *p* = 0.006) (Supplementary Figure S1C). Increases in ʃGG _EMG_ activity and respiratory rate did not significantly differ from direct PAG stimulation (*F* (2,25) = 0.46, *p* = 0.640).

When we specifically assessed the efficacy of bilateral versus unilateral stimulation of PAG terminals in preBötC we found some interesting differences. The median effects for the inspiratory muscle activities and respiratory rate were lower in the unilateral case compared to the bilateral group (∫DIA_EMG_ = 1.20 vs 1.05; ∫GG = 1.33 vs 1.20; Resp. Rate: 1.24 vs 1.16) (Supplementary Figure S1C-E). Despite these differences, post-hoc testing was unable to distinguish a significant difference between evoked ∫DIA_EMG_ activity following bilateral versus unilateral stimulation protocols (Tukey HSD: *p* = 0.166), nor any significant differences when comparing the genioglossal activities and respiratory rates (*F* (2,25) = 2.21, *p* = 0.130) (Supplementary Figure S1D, E).

Lastly, photostimulation targeted at terminals in the preBötC did not evoke any notable changes in ∫ABD_EMG_ activity in either bilateral or unilateral experiments. Relative ∫ABD_EMG_ activity remained at the level of natural breathing when bilaterally stimulating PAG fibres in the preBötC (median = 1.00, *t* (7) = −1.77, *p* = 0.120), which was also significantly lower compared to direct stimulation of the PAG (*H* (2,27) = 19.71, *p* < 0.001, Dunn: *p* < 0.001) (Figure 6F). Similarly, abdominal activity following unilateral stimulation in the preBötC did not significantly differ from baseline (median = 1.01, *t* (4) = 1.48, *p =* 0.213) and was significantly lower than direct PAG stimulation (*H* (2,25) = 19.71, *p <* 0.001, Dunn: *p* = 0.032; Supplementary Figure S1D).

#### pFL stimulation

We next investigated whether the prominent abdominal activation we observed in response to PAG stimulation was mediated by direct projections to the pFL (Biancardi et al., 2021), which is critically implicated in the generation of forced expiratory activity. Bilateral stimulation of the pFL with 10 s pulses or high-frequency pulse trains (n = 7) did not elicit any significant changes in diaphragmatic muscle activity in our experiments (Figures 6C,D: ∫DIA_EMG_: median = 1.03, *w* = 22, *p* = 0.219). Stimulation in this area did evoke subtle increases in ʃGG _EMG_ activity as well as mild increases in respiratory rate which were followed in some experiments by respiratory rate slowing (Figures 6C,D). These subtle increases reached a median of ∼1.2 and ∼1.1 for relative ∫GG_EMG_ (*w* = 28 *p* = 0.016) and relative respiratory rate respectively (*t* (6) = 4.20, *p* = 0.006) (Figures 6E,G). While these effects did not significantly differ from those elicited by bilateral stimulation in the preBötC (∫GG_EMG_: *H* (2,27) = 1.87, *p* = 0.392; RR: *F* (2,27) = 4.92, *p* = 0.015, Tukey HSD: *p* = 0.254), they tended towards lower effect sizes (Figures 6E,G). Moreover, the increase in respiratory rate following direct stimulation of the PAG was significantly greater than that following bilateral stimulation in the pFL (Tukey HSD: *p* = 0.012), but not the preBötC as detailed above. Stimulating PAG projections in the pFL was unable to produce significant changes in ∫ABD_EMG_ relative to baseline breathing (∫ABD_EMG_: median = 1.00, *t* (6) = −0.198, *p* = 0.850) (Figure 6F), suggesting that in our experimental conditions stimulation of PAG terminals in the pFL is not sufficient to generate active expiration.

### Brief pulse stimuli targeting PAG terminals in preBötC and pFL do not produce phase-dependent changes in respiratory timing or activity

In order to elucidate the contributions of key brainstem respiratory regions in the effects of brief PAG activation on respiratory cycle timings, we conducted the same experimental paradigm as described above for direct PAG stimulation with short laser pulses, instead targeting PAG projections in key downstream areas. Given our results for unilateral and bilateral stimulation did not differ, we combined these datasets for the following analyses.

#### preBötC stimulation

**FIGURE 7:**
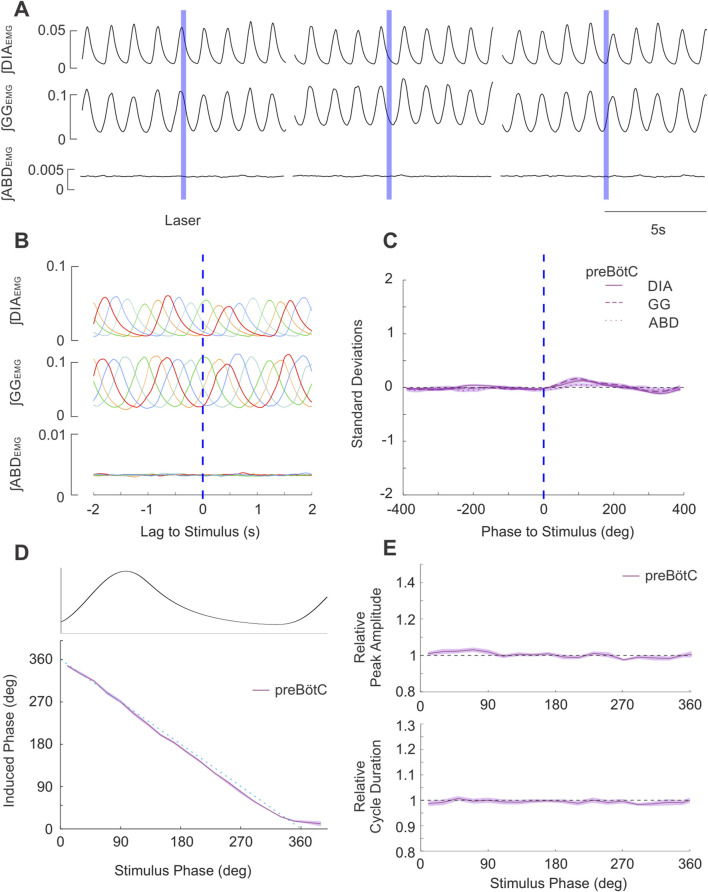
Brief stimulation of PAG terminals in preBӧtC does not elicit changes in respiratory EMG activities nor changes in respiratory timing. **(A)** Example integrated EMG traces for three different instances of brief (250 ms) pulse stimulation (blue boxes) delivered at different phases of the natural ongoing breathing rhythm. No apparent alteration in the rhythmic expression of EMG activities are observed. **(B)** Stimulus-triggered overlay of integrated EMG activity. Five breathing cycles during which stimulus pulses arrived at different phases of the ongoing respiratory cycle are overlaid and time-locked to the onset of laser pulses (lag = 0s). No stimulus locking of subsequent rhythmic EMG is apparent. **(C)** Stimulus-triggered average for all experiments. Integrated EMG activities (see legend) were normalized in both time/phase and amplitude and aligned to the onset of stimulus (0 lag, dashed blue line) and averaged to highlight the lack of locked responses post-stimulation across all experiments (n = 20). Shaded areas indicate ± S.E.M. **(D)** Stimulus pulses arriving at different phase of the respiratory cycle (aligned and overlaid above D) failed to evoke changes in the induced phase (purple relative to the expected phase (dashed blue). Shaded area indicates ± S.E.M. **(E)** (top): Amplitude of the peak ∫DIA_EMG_ activity elicited by stimulation at different respiratory phases relative to the ∫DIA_EMG_ peak of natural cycles (dashed line at 1). (bottom): Same as top, duration of the cycle following stimulus pulses relative to duration of natural breaths.

As demonstrated in Figure 7A, brief stimulation in the preBötC (n = 20) did not produce any effects on the timing of the respiratory cycle. Moreover, we did not observe any effects on the ongoing respiratory rhythm for any stimulus phase (Figure 7B). In contrast to the results following direct PAG stimulation, brief laser pulses did not elicit stimulus-locked responses in either inspiratory or expiratory muscle activity (Figure 7C). Systematic analysis of cycle timing via the methods detailed in the results above for direct stimulation revealed that the induced phase very closely followed the linear expected phase relationship with stimulus phase (Figure 7D). Furthermore, our analysis of evoked amplitude and cycle duration indicated there was no facilitation of ∫DIA_EMG_ (Figure 7E) or ∫GG_EMG_ amplitude, nor any substantial increases in cycle duration relative to natural breathing (Figure 7E).

#### pFL stimulation

**FIGURE 8:**
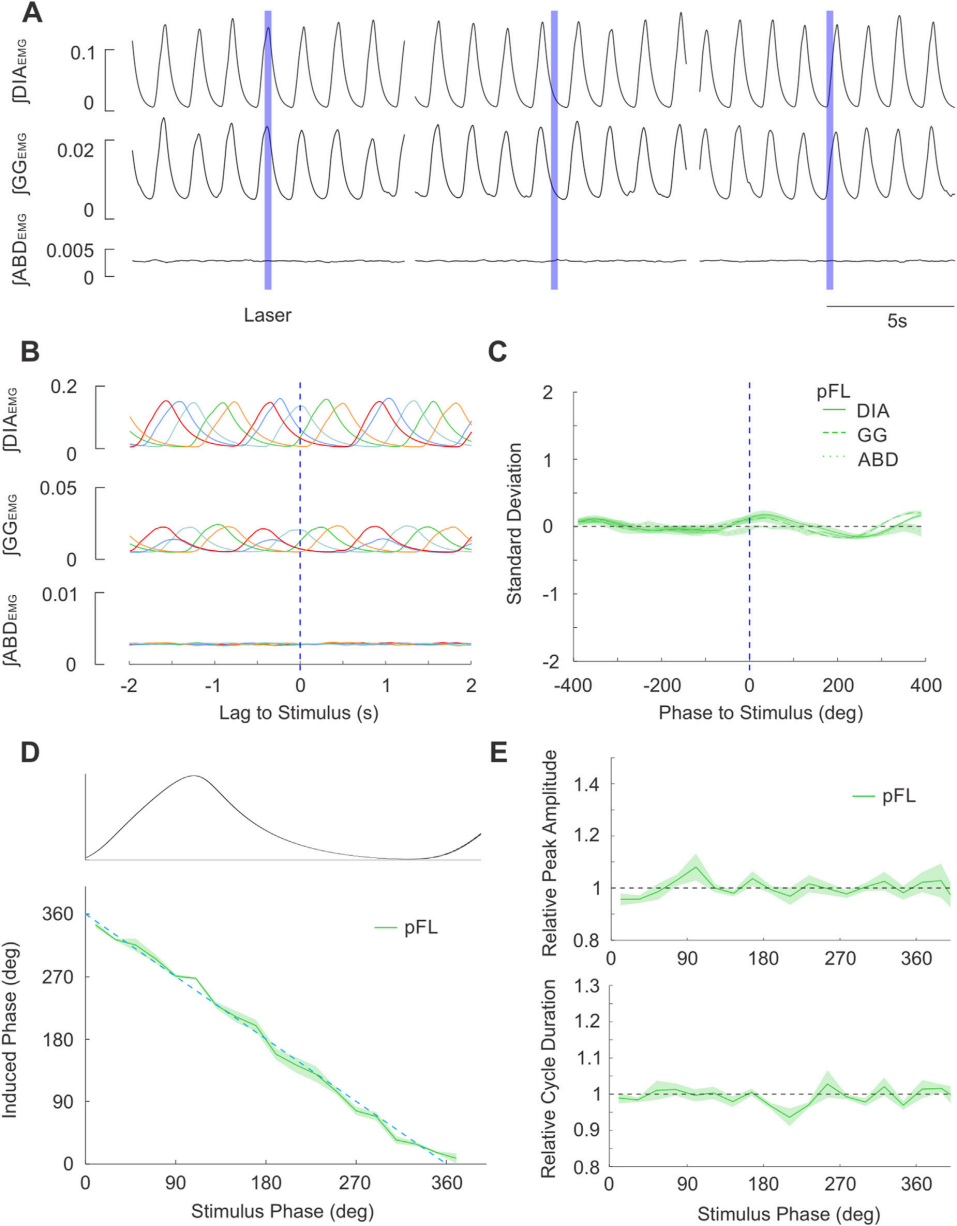
Brief stimulation of PAG terminals in pFL does not elicit changes in respiratory EMG activities nor changes in respiratory timing. **(A)** Example integrated EMG traces for three different instances of brief (250 ms) pulse stimulation (blue boxes) delivered at different phases of the natural ongoing breathing rhythm. No apparent alteration in the rhythmic expression of EMG activities are observed. **(B)** Stimulus-triggered overlay of integrated EMG activity. Five breathing cycles during which stimulus pulses arrived at different phases of the ongoing respiratory cycle are overlaid and time-locked to the onset of laser pulses (lag = 0s). No stimulus locking of subsequent rhythmic EMG is apparent. **(C)** Stimulus-triggered average for all experiments. Integrated EMG activities (see legend) were normalized in both time/phase and amplitude and aligned to the onset of stimulus (0 lag, dashed blue line) and averaged to highlight the lack of locked responses post-stimulation across all experiments (n = 4). Shaded areas indicate ± S.E.M. **(D)** Stimulus pulses arriving at different phase of the respiratory cycle (aligned and overlaid above D) failed to evoke changes in the induced phase (green) relative to the expected phase (dashed blue). Shaded area indicates ± S.E.M. **(E)** (top): Peak amplitude of ∫DIA_EMG_ activity elicited by stimulation at different respiratory phases relative to the peak during natural cycles (dashed line at 1). (bottom): Same as top, cycle duration following stimulus pulses relative to duration of natural breaths.

Similarly, brief bilaterally targeted pulses into the pFL (n = 4) did not produce any consistent effects on respiratory activity or timing (Figures 8A–C). Induced phase followed the expected linear relationship with stimulus phase, indicating there was no significant resetting of the respiratory rhythm (Figure 8D). We did not observe any substantial changes in ∫DIA_EMG_ (Figure 8E) or ∫GG_EMG_ amplitude and cycle duration relative to baseline.

## Discussion

This study investigated the respiratory responses obtained through optogenetic stimulation of the PAG neurons in urethane-anesthetized, vagotomized, and spontaneously breathing adult rats. Based on our histological results, the area of activation included the lateral PAG and the more dorsal extension of the ventrolateral PAG. Similar to previous studies with chemical and electrical stimulations within this area, we observed an increase in respiratory rate during PAG photostimulation, associated with an increased in peak amplitude of both inspiratory ∫DIA and ∫GG_EMG_ activity, as well as activation of expiratory modulated ∫ABD activity. Interestingly, we observed a fine tuning of ongoing respiratory rhythm that consisted of respiratory reset, where brief PAG photostimulation pulses delivered during the inspiratory phase enhanced the ongoing breath (with increase in peak ∫DIA and ∫GG_EMG_ activity) and expanded the relative cycle duration. When brief photostimulation pulses were delivered during the post-inspiratory phase as well as during expiration, they triggered a consistent phase advance of the subsequent respiratory cycle without significant alteration of the peak amplitude of respiratory muscle activation. We further investigated whether this effect was due to direct projections to the rhythmogenic areas of the preBötC and/or pFL by attempting to induce a similar respiratory reset by stimulation of PAG presynaptic terminals in the preBötC and pFL, the inspiratory and expiratory oscillators, respectively. Despite some very modest effects on respiratory frequency following high frequency stimulation in the preBötC, respiratory reset was not elicited with short pulses in either location, suggesting that PAG neurons may act to reset ongoing breathing through an extended and distributed network that in turn affects rhythmogenic centers.

### Stimulation of PAG enhances respiratory activity

The PAG is implicated in multiple integrative functions that are tightly associated with changes in respiration (Benarroch, 2012; Bandler and Shipley, 1994; Faull et al., 2019; Tovote et al., 2016; Dampney, 2015; Samineni et al., 2019). Chemical stimulation of different PAG subregions generate distinct respiratory responses that are associated with the flight or fight response, emotion, pain, and vocalization mediated by the projections from various forebrain structures (Zhang et al., 2024). The elicited respiratory responses vary from apnea generation, bradypnea, irregular breathing, tachypnea, and they have been attributed to the activation of distinct PAG neurons that project to the respiratory control network in the pons and the medulla affecting the activity of inspiratory, post-inspiratory and expiratory neurons (Huang et al., 2000; Zhang et al., 2005; Subramanian et al., 2008; Iigaya et al., 2010; Faull et al., 2019; Farmer et al., 2014; Subramanian, 2013).

Although the neuronal pathways responsible for these respiratory effects are currently unknown, anatomical tracing studies indicate that PAG neurons project to the nucleus retroambiguus, the Kölliker Fuse, the locus coeruleus and raphe nuclei, but also neurons within the ventral medulla, including the parasympathetic neurons in the nucleus ambiguus, the pain circuitry in the rostral ventromedial medulla, the sympathetic neurons of the rostral ventrolateral medulla, as well as respiratory neurons of the ventral respiratory column (BötC, preBötC, pFV and pFL) (Trevizan-Baú et al., 2021b; Biancardi et al., 2021; Ennis et al., 1997; Yang et al., 2020; Rosin et al., 2006; Matsuyama and Horiuchi, 2024; Harding et al., 2024; Dempsey et al., 2017). On the contrary, the dorsolateral PAG displays a unique set of afferent and efferent projections that possibly differ from both the adjacent dorsal and lateral subdivisions of the PAG and may have a unique role in integrating cardiovascular and respiratory responses to psychological stressors (Trevizan-Baú et al., 2021b; Biancardi et al., 2021; Ennis et al., 1997; van Bockstaele et al., 1991; Dampney, 2015; Yang et al., 2020; Rosin et al., 2006).

Our stereotaxic injections aimed to target the lateral PAG subdivision and histological verification indicates that the core of both viral and induced cFos expression following repeated stimulations was in the lateral and ventrolateral PAG with a modest diffusion into the adjacent portions of the dorsolateral PAG. Moreover, the observed increases in breathing frequency as well as the increased inspiratory and expiratory muscle activity following a long pulse stimulation of infected PAG neurons, is in accordance with the tachypneic and respiratory effects observed with localized lateral PAG chemical stimulation (Subramanian et al., 2008; Iigaya et al., 2010; Farmer et al., 2014; Subramanian, 2013).

In a subset of rats, we investigated the relative activation of cells in various areas involved in respiratory control following repeated PAG pulse stimulation, as it could give us an indication of the pathways that were activated during stimulation. These results showed that an increased number of cFos expressing cells was observed in several brainstem areas within the respiratory network: the LC, the reticular formation, and the ventral respiratory column, specifically the pFL, BötC, and preBötC areas. Interestingly, we observed no difference in cFos expression compared to control experiments at the level of the parabrachial nuclei, the NTS, the pFV (which includes the RTN) or, surprisingly, the more caudal region of the respiratory column containing inspiratory and expiratory premotoneurons (rVRG and cVRG) despite the presence of GFP + fibers as well as well established anatomical connections (Zhang et al., 2024; Trevizan-Baú et al., 2021b; Lima et al., 2018; Chen et al., 2022; Farkas et al., 1997). While PAG projections to the LC have been recently proposed to be involved in nociceptive pathways (Lubejko et al., 2024), and the projections to NTS are involved in autonomic and cardiovascular regulation (Chen et al., 2022; Wilson and Kapp, 1991) but also respiration (Huang et al., 2000; Subramanian et al., 2007a), the projections to pFL, BötC, and preBötC may be directly involved in respiratory modulation, as suggested in previous work (Zhang et al., 2024; Trevizan-Baú et al., 2021b).

These results suggest preferential effects of activation of selected regions following our experimental protocol that we explored further in the subsequent experiments, although we cannot exclude that our cFos staining is not sufficient to identify all cells activated by PAG stimulation.

### Pulse stimulation of PAG resets respiratory rhythm with an inspiratory-driven phase dependent response

Our chosen optogenetic approach allowed us to deliver a localized and time-controlled stimulation within the respiratory cycle which revealed potent phase-dependent effects on the ongoing respiratory cycle. Stimulation of the PAG results in a new breath being drawn after a short delay, after which the timing of inspiratory (diaphragmatic and genioglossal) muscle activities is conserved. Maintaining the pattern of muscle contractions in this way may be relevant for the function of naturalistic PAG-related modulation of breathing, as a sharp intake of breath following an emotionally salient event needs to retain coordination of the upper and lower airways for proper air intake. Moreover, this result demonstrates that PAG activation is sufficient to drive respiratory outputs in a precisely timed manner.

Several phase-dependent effects were observed as photostimulation during the inspiratory phase potentiated the inspiratory effort (enhanced ∫DIA_EMG_ amplitude) and delayed the subsequent inspiratory bout resulting in an increased cycle duration. Conversely, breaths elicited by stimulation in the expiratory period did not exhibit any increased amplitude and caused a phase reset of the respiratory rhythm, with a delay which was often dependent on the phase at which stimulation was delivered and varied in a sigmoidal relationship.

The delayed inspiratory burst following inspiratory stimulation also manifested as an induced phase which was on average ∼10° beyond a full cycle length (∼370°, Figure 4). We note that while our chosen EMG-based methodology prevents an accurate assessment of the true induced delay of inspiratory-related bursts of neural activity in the preBӧtC, the similarity between this ∼10° delay and the ∼10° delay induced by stimulation during the late expiratory period suggests that our stimulation did indeed reset the respiratory rhythm even during inspiration (Figure 4).

The similarity between the lengthened cycle and phase delay noted during the stimulation in the inspiratory and expiratory phases respectively, was particularly clear only in a subset of experiments (∼28%) in which induced phase followed a step-like function (Figure 4F). Given the comparable delays mentioned above and the sharp transition between the inspiratory and expiratory plateaus, the reset observed in these experiments may be classified as a phase-independent, or “type 0” reset (Alsahafi et al., 2015; Winfree, 2001). Moreover, this response matches those observed in previous experiments when stimulating the preBӧtC network directly (Alsahafi et al., 2015). While our virus targeted neurons irrespective of their neuronal phenotype with a synapsin promoter, the lateral/ventrolateral PAG contains mostly glutamatergic projecting neurons and a local network of GABAergic interneurons (Benarroch, 2012; Tovote et al., 2016; Reichling et al., 1991), unpublished results), suggesting that stimulation to preBötC are for the most part glutamatergic and excitatory. Although we did not characterize the relative expression of the optogenetic virus within the glutamatergic and GABAergic cells, nor did we perform a systematic comparison of infected cells, the extent of the viral spread and location of the optic fibers (as shown in Figure 1) was similar across subjects and would not account for the different types of reset that our stimulation produced. The differences in our dataset may therefore reflect subtle variability in the neuronal population infected by the virus and their projections to target regions that we were unable to detect. Alternatively, the existence of both phase-dependent and independent types of reset response may indicate a state-dependent relationship in which the post-inspiratory delay is shortened leading to the sharp, step-wise transition in the minority of our experiments. Further experiments which record the strength of elicited neural activity in downstream target regions as well as the cell types involved may also help explain the variability in the reset response we observed here.

In order to determine whether the respiratory reset was caused by a direct effect on the PAG-preBötC projections ((Trevizan-Baú et al., 2021b; Yang et al., 2020), current results) we unilaterally and bilaterally stimulated preBötC. We selected unilateral photostimulation on the ipsilateral side as PAG projections to the preBötC are predominantly ipsilateral to the injection site (Trevizan-Baú et al., 2021b). Although we observed in both cases an increase in respiratory rate and in peak ∫DIA and ∫GG_EMG_ amplitude (with an enhanced effect observed with bilateral stimulation), this approach did not cause any significant respiratory reset response. Although this may be caused by the reduced effect of opsin activation at presynaptic terminals compared to the soma/axon hillock activation of infected PAG neurons, we propose that the PAG stimulation-induced respiratory reset requires an indirect pathway or, more likely, a more distributed activation of the respiratory network to reset preBötC activity, as previously suggested (Trevizan-Baú et al., 2021b). Tracing studies showed that lateral PAG has extensive reciprocal projections with the KF nucleus, as well as major projections to the nucleus retroambiguus, the raphe nuclei and the preBötC, (Zhang et al., 2024; Trevizan-Baú et al., 2021b), thus supporting the stimulatory effects observed with preBötC area stimulation. Intense projections GFP + projections were observed in the PBN although no changes in cFOS activation compared to control would suggest an increased activity of this areas. Furthermore, PAG projections, as well as cFos activation in the intermediate reticular formation and the LC suggest the possibility that these areas could be involved in respiratory excitation. It will be important to test their role in promoting respiratory reset with PAG terminal stimulation in these areas or with intersectional optogenetic approaches in future experiments.

### Stimulation of PAG activate expiratory abdominal activity independent of pFL activation

Previous work showed that PAG chemical stimulation activates expiratory abdominal activity (Subramanian et al., 2008; Subramanian and Holstege, 2014), possibly via activation of the expiratory premotoneurons in the nucleus retroambiguus/cVRG (Holstege and Subramanian, 2016) or through identified anatomical projections to the pFL (Biancardi et al., 2021). In our experiments we only observed abdominal activation when stimulation was delivered with long trains of excitation to the PAG and it was absent following brief pulses of PAG stimulation that triggered the observed inspiratory driven response. Similarly, the recruitment of ABD muscle activity was not observed with direct pFL stimulation, suggesting that PAG terminal stimulation in the pFL may not be sufficient to promote pFL-driven active expiration. In contrast to direct pFL stimulation (Pagliardini et al., 2011), we never observed interruption of the ongoing inspiratory cycle or induction of an expiratory driven reset with PAG terminal stimulation at the level of pFL. Because the reset observed with soma PAG stimulation was consistently inspiratory-driven even during the expiratory phase, we propose that recruitment of ABD activity bypasses pFL and it is driven by the stimulation of expiratory premotor neurons in the cVRG either through direct activation of these neurons or through indirect stimulation of the distributed ponto-medullary respiratory network.

In summary, these results support the key role of PAG in fine tuning ongoing respiratory rhythm that would be relevant for behaviours associated with enhanced ventilation during pain, emotional control, vocalization, and orofacial behaviours. Such a strategy would involve a diverse set of respiratory related centers in the brainstem, including the KF, the NRA, the NTS, the LC, caudal raphe, working together to alter breathing during such behaviours (Jürgens, 2002; Trevizan-Baú et al., 2021b; Subramanian et al., 2008; Vanderhorst et al., 2000; Subramanian et al., 2007b). Further experimentation is needed, however, to determine the exact contribution each area makes to these vital functions as well as the time course of their involvement. Future investigation which aims to record neural activity in the preBötC, KF, or pFL over the course of PAG stimulation would give valuable insight into how respiratory rhythm generators are influenced during the volitional behaviors and emotional states signaled by PAG activity. This is of particular interest given the complex arrangement of reciprocal connections made within the brainstem respiration centers noted above as well as those recently noted between the PAG and downstream respiratory-related targets (Zhang et al., 2024; Trevizan-Baú et al., 2021b).

## Data Availability

The raw data supporting the conclusions of this article will be made available by the authors, without undue reservation.
