# Supplementary figures and images for: Activation of the periaqueductal gray controls respiratory output through a distributed brain network
Source: Front Physiol. 2025 Jan 22;16:1516771. doi: 10.3389/fphys.2025.1516771 (PMC11794281; doi:10.3389/fphys.2025.1516771)

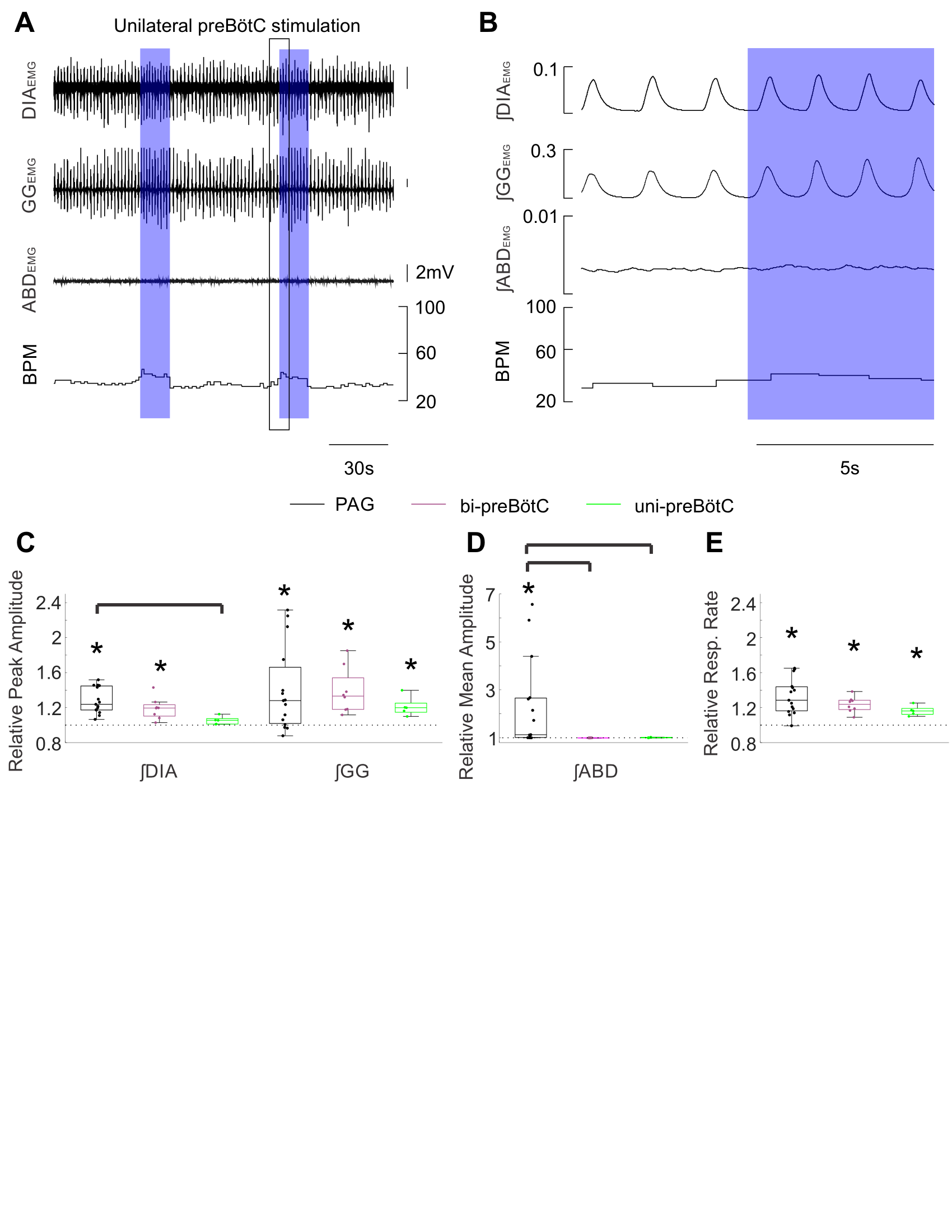

Supplement: Supplementary file 1 [file Image1.tiff]
