# Supplementary material for: Activation of the periaqueductal gray controls respiratory output through a distributed brain network
Source: Front Physiol. 2025 Jan 22;16:1516771. doi: 10.3389/fphys.2025.1516771 (PMC11794281; doi:10.3389/fphys.2025.1516771)
Supplement: Supplementary file 2 [file DataSheet1.docx]

**Supplemental Figure 1: Unilateral stimulation of PAG terminals in preBӧtC produces weaker, but significant responses compared to bilateral stimulation**. **A**: Example recording of raw diaphragm (DIA), genioglossus (GG) and abdominal (ABD) _EMG_ activity alongside respiratory rate (breath per minute, BPM) following bursts of photostimulation (blue boxes). **B**: Integrated and magnified activity and rate recordings from the black box in A. **C**: Peak amplitude of ʃDIA and ∫GG_EMG_ activity following stimulation in the PAG (black, n = 15), bilateral (purple = 8), and unilateral (green, n = 5) stimulation of PAG terminals in preBӧtC relative to peak amplitude during baseline (dashed line at 1). Dots correspond to individual experiments. Boxes indicate the middle quartiles and median (line), whiskers denote outer quartiles. Stars mark significant differences from 1 at the level of p = 0.05 as per t-test or sign test. Brackets indicate significant difference between groups at p = 0.05 as determined by Kruskal-Wallis with a Dunn’s test or 1way AVOVA with Tukey’s HSD post-hoc test. **F**: Similar to E, mean amplitude of ∫ABD_EMG_ activity during stimulation relative to mean baseline activity (dashed line at 1). **G**: Similar to E, mean respiratory rate during stimulation relative to baseline.
